# Author Correction: Anthropogenic climate and land-use change drive short- and long-term biodiversity shifts across taxa

**DOI:** 10.1038/s41559-024-02405-9

**Published:** 2024-03-28

**Authors:** Teresa Montràs-Janer, Andrew J. Suggitt, Richard Fox, Mari Jönsson, Blaise Martay, David B. Roy, Kevin J. Walker, Alistair G. Auffret

**Affiliations:** 1 Department of Ecology, Swedish University of Agricultural Sciences, Uppsala, Sweden; 2 Department of Geography and Environmental Sciences, Northumbria University, Newcastle, UK; 3 Butterfly Conservation, East Lulworth, UK; 4 Swedish Species Information Centre, Swedish University of Agricultural Sciences, Uppsala, Sweden; 5 British Trust for Ornithology, Thetford, UK; 6 UK Centre for Ecology & Hydrology, Wallingford, UK; 7 Botanical Society of Britain and Ireland, Harrogate, UK

**Keywords:** Biodiversity, Conservation biology, Climate-change ecology

Correction to: *Nature Ecology & Evolution* 10.1038/s41559-024-02326-7, published online 12 February 2024.

In the version of the article initially published, the “Baseline conditions” in Fig. 3a were incorrectly ordered. The list originally read “Arable”, “Improved grasslands”, “Forest”, “Urban”, “Temperature”, “Precipitation” and “Seminatural grasslands” but has now been amended to “Semi-natural grasslands”, “Arable”, “Improved grasslands”, “Forest”, “Urban”, “Temperature” and “Precipitation” in the HTML and PDF versions of the article.

