# Supplementary material for: Anthropogenic climate and land-use change drive short- and long-term biodiversity shifts across taxa
Source: Nat Ecol Evol. 2024 Feb 12;8(4):739–51. doi: 10.1038/s41559-024-02326-7 (PMC11009105; doi:10.1038/s41559-024-02326-7)
Supplement: Supplementary file 1 — Supplementary Tables 1–5, Figs 1–8 and Notes. [file 41559_2024_2326_MOESM1_ESM.pdf]

nature ecology & evolution

Article https://doi.org/10.1038/s41559-024-02326-7

Anthropogenic climate and land-use change drive short- and long-term biodiversity shifts across taxa

In the format provided by the authors and unedited

Supplementary information

Anthropogenic climate and land-use change drive short and long-term biodiversity shifts across taxa

Teresa Montràs-Janer et al.

Supplementary Tables

Supplementary Table 1. Correlations between the observed percentage of number of species gained and lost.

Supplementary Table 2. Associations between changes in species richness, beta diversity and community temperature index and changes in land use and climate.

Supplementary Table 3. Fixed effects and 95% credible interval (95% CI) for the associations between local contribution to beta diversity and land use and climate in Great Britain.

Supplementary Table 4. Fixed effects (95% credible interval) for the changes of species richness, beta diversity and community temperature index for three different taxon communities in Great Britain.

Supplementary Table 5. Comparison of predicted values of changes in species richness (SR), beta diversity (BD) and community temperature index (CTI) using different model structures.

Supplementary Figures

Supplementary Fig. 1. Land use and climate in Great Britain at three different time periods

Supplementary Fig. 2. Consistency of the change models estimating fixed effects

Supplementary Fig. 3. Associations between interacting effects of land-use and climate change and biodiversity change

Supplementary Fig. 4. Comparison of R-square marginal and R-square conditional

Supplementary Fig. 5. Consistency of the LCBD models estimating fixed effects

Supplementary Fig. 6. Short-term biodiversity change for the British butterflies

Supplementary Fig. 7. Estimated recorder effort for the British breeding birds, British butterflies, and British plants atlases at different time periods

Supplementary Fig. 8. Associations between change in biodiversity and change in and baseline of environmental variables

Supplementary Notes

Taxonomic filtering of observation data

The iCAR model structure

Dealing with collinearity

Assessing spatial dependency

Assessing model compatibility

Penalised Complexity priors

Supplementary Table 1. Correlations between the observed percentage of number of species gained and lost for three different taxon communities in Great Britain at the long (1960s to 2010s) and short-term (1990s to 2010s), and the baseline conditions (as described in Methods) of species richness, beta diversity and semi-natural grasslands. The numbers indicate the Pearson correlation coefficient (two-sided). Only correlations with p-values < 0.05 are shown. All correlations P<0.001 aside from those marked with ** denoting P<0.01. Note that higher baseline levels of species richness and lower beta diversity are associated with more stable communities over time, i.e., lower percentages of both species gained and lost.

	BIRDS				BUTTERFLIES				PLANTS			
	LONG		SHORT		LONG		SHORT		LONG		SHORT	
Baseline	Prop. Gain	Prop. Loss	Prop. Gain	Prop. Loss	Prop. Gain	Prop. Loss	Prop. Gain	Prop. Loss	Prop. Gain	Prop. Loss	Prop. Gain	Prop. Loss
n cells	*2670*	*2670*	*2670*	*2670*	*2013*	*2013*	*2022*	*2022*	*2666*	*2666*	*2666*	*2666*
Species richness	-0.624	-0.182	-0.459		-0.568	-0.118	-0.591	-0.421	-0.217		-0.092	
Beta diversity	0.557	0.377	0.535	0.279	0.638	0.122	0.513	0.229	0.547	0.190	0.272	
Semi-nat. grasslands	0.063	0.255	0.118	0.299	0.044**	0.177	0.268	0.434		-0.067	0.055**	

Positive association Negative association No association

Supplementary Table 2. Associations between changes in species richness, beta diversity and community temperature index and changes (Ch.) of land use and climate, interactions between changes of land use and climate, baseline conditions of land use and climate, baseline biodiversity (for richness and beta diversity) and microclimate heterogeneity, for three different taxon communities in Great Britain over the long (1960s - 2010s) and short-term (1990s - 2010s). Estimates derived from spatially-explicit linear mixed-effects models with Gaussian distribution, see on model eq. 4 for details (Methods). Colored bars represent the fixed effect value of each explanatory variable over the response *biodiversity change*: the higher the value, the longer the bar (Supplementary Fig. 8 for details in estimated curves). Values of R-square conditional and marginal are given for each model - underlined values indicate that the model incorporates spatial dependency. White cells mean that no associations were found.

SPECIES RICHNESS

		BIRDS		BUTTERFLIES		PLANTS	
		long	short	long	short	long	short
	Intercept	1,733	1,633	10,262	6,901	49,158	-6,209
**Interactions environmental change**	Ch.Arable:Ch.Temp	0,767					
	Ch.Arable:Ch.Prec			-0,567			
	Ch.Imp.grass:Ch.Temp						
	Ch.Imp.grass:Ch.Prec					8,438	
	Ch.Forest:Ch.Temp						-4,162
	Ch.Forest:Ch.Prec	-0,446		-0,225			
	Ch.Urban:Ch.Temp					3,551	
	Ch.Urban:Ch.Prec	0,776				10,779	
**Environmental change**	Ch. Arable	-0,685	-0,721	1,632		7,201	-5,161
	Ch. Improved grasslands			1,115		17,453	-5,025
	Ch. Forest	0,662		1,083		8,862	
	Ch. Urban	0,684		0,482		10,893	
	Ch. Temperature			0,559			-3,009
	Ch. Precipitation				0,327	13,914	2,974
**Baseline conditions**	Semi-natural grasslands		-1,344	-1,556	-2,253	-10,282	-11,614
	Arable		1,183	1,279	1,791		
	Improved grasslands		1,909		1,196		8,885
	Forest	1,073	1,771	1,465	1,485	10,693	8,892
	Urban		1,181	1,208	1,427	7,607	7,119
	Temperature			0,701	1,033	4,953	
	Precipitation	-1,470	-1,291				
	Biodiversity	-4,573	-6,320	-5,790	-5,178	-51,238	-33,711
	Microclimate heterogeneity			0,507	0,720	5,382	4,606
	R-square (conditional)	0,61	0,76	0.80	0,85	0,54	0,46
	R-square (marginal)	0,27	0,41	0,55	0,50	0,54	0,46

BETA DIVERSITY

		BIRDS		BUTTERFLIES		PLANTS	
		long	short	long	short	long	short
	Intercept	0,007	-0,021	-0,335	-0,237	-0,039	-0,001
**Interactions environmental change**	Ch.Arable:Ch.Temp						
	Ch.Arable:Ch.Prec	-0,004		0,012			
	Ch.Imp.grass:Ch.Temp	0,003		0,009			0,005
	Ch.Imp.grass:Ch.Prec						
	Ch.Forest:Ch.Temp			0,008			
	Ch.Forest:Ch.Prec						
	Ch.Urban:Ch.Temp					-0,005	
	Ch.Urban:Ch.Prec						
**Environmental change**	Ch. Arable	-0,005		-0,027			
	Ch. Improved grasslands	-0,004	0,004	-0,029		-0,007	0,004
	Ch. Forest	-0,003		-0,009	0,009	-0,007	-0,007
	Ch. Urban			-0,013			
	Ch. Temperature	-0,003		-0,009	-0,008		
	Ch. Precipitation			-0,013	-0,011		
**Baseline conditions**	Semi-natural grasslands		0,010	0,027	0,032		
	Arable		-0,010	-0,010	-0,018	0,004	0,005
	Improved grasslands		-0,009		-0,025		
	Forest		-0,007	-0,008	-0,018		
	Urban		-0,003	-0,012	-0,021		0,005
	Temperature			-0,018	-0,033		0,006
	Precipitation	0,003			-0,013	-0,006	
	Biodiversity	-0,026	-0,029	-0,210	-0,204	-0,069	-0,037
	Microclimate heterogeneity						
	R-square (conditional)	0,52	0,64	0,82	0,86	0.70	0,46
	R-square (marginal)						

COMMUNITY TEMPERATURE INDEX

		BIRDS		BUTTERFLIES		PLANTS	
		long	short	long	short	long	short
	Intercept	0,039	0,123	0,015	-0,024	0,070	0,062
**Interactions environmental change**	Ch.Arable:Ch.Temp		0,010	-0,013			
	Ch.Arable:Ch.Prec				0,016		
	Ch.Imp.grass:Ch.Temp		0,010	-0,020			
	Ch.Imp.grass:Ch.Prec						
	Ch.Forest:Ch.Temp						
	Ch.Forest:Ch.Prec						
	Ch.Urban:Ch.Temp		0,009		0,013		
	Ch.Urban:Ch.Prec	-0,021					
**Environmental change**	Ch. Arable				0,014		
	Ch. Improved grasslands						
	Ch. Forest	0,013					
	Ch. Urban	-0,018	-0,010				
	Ch. Temperature						
	Ch. Precipitation						
**Baseline conditions**	Semi-natural grasslands		0,015				
	Arable				-0,025	0,011	0,011
	Improved grasslands		0,011				0,013
	Forest						0,008
	Urban				-0,020	0,011	0,015
	Temperature				0,016		-0,010
	Precipitation						
	Biodiversity						
	Microclimate heterogeneity	-0,027	-0,024				
	R-square (conditional)	0.60	0,42	0,11	0.45	0,61	0,68
	R-square (marginal)	0,12	0,03	0,06	0,06	0,09	0,05

Positive associations

Negative associations

Supplementary Table 3. Fixed effects and 95% credible interval (95% CI) for the associations between local contribution to beta diversity and land use and climate in Great Britain, based on model eq. 5 (Methods), for three different taxon communities and three different time periods (1960s, 1990s and 2010s). Grey blocks indicate that there was no improved grassland category in the 1960s land-cover dataset. White blocks mean that no associations were found.

BIRDS

	1960s		1990s		2010s	
	Mean	95% CI	Mean	95% CI	Mean	95% CI
Intercept	-3.324	(-3.335 , -3.312	-3.321	(-3.330 , -3.312)	-3.323	(-3.332 , -3.314)
Semi-natural grasslands	0.103	(0.084 , 0.122)	0.298	(0.280 , 0.317)	0.325	(0.306 , 0.343)
Arable	-0.081	(-0.095 , -0.067)	-0.096	(-0.115 , -0.077)	-0.114	(-0.134 , -0.094)
Improved grasslands			-0.082	(-0.103 , -0.062)	-0.073	(-0.094 , -0.051)
Forest	-0.137	(-0.154 , -0.121)	-0.097	(-0.111 , -0.083)	-0.104	(-0.120 , -0.089)
Urban	-0.066	(-0.084 , -0.048)	-0.043	(-0.058 , -0.028)	-0.040	(-0.055 , -0.024)
Temperature	0.043	(0.020 , 0.067)	0.053	(0.032 , 0.074)	0.068	(0.044 , 0.092)
Precipitation	0.039	(0.013 , 0.064)	0.031	(0.007 , 0.056)	0.037	(0.008 , 0.066)
Microclimate heterogeneity			-0.048	(-0.066 , -0.029)	-0.049	(-0.069 , -0.030)

BUTTERFLIES

	1960s		1990s		2010s	
	Mean	95% CI	Mean	95% CI	Mean	95% CI
Intercept	-2.977	(-2.993 -2.961)	-3.056	(-3.070 , -3.043)	-3.099	(-3.110 , -3.087)
Semi-natural grasslands	0.028	(0.003 0.054)	0.145	(0.116 , 0.174)	0.276	(0.250 , 0.302)
Arable	-0.054	(-0.084 -0.024)			-0.089	(-0.123 , -0.055)
Improved grasslands						
Forest	-0.050	(-0.073 -0.027)	-0.030	(-0.055 , -0.004)	-0.039	(-0.065 , -0.013)
Urban	-0.063	(-0.092 -0.034)	-0.046	(-0.078 , -0.014)	-0.067	(-0.098 , -0.036)
Temperature	-0.035	(-0.064 -0.006)			-0.095	(-0.130 , -0.059)
Precipitation			0.054	(0.017 , 0.092)	-0.049	(-0.088 , -0.010)
Microclimate heterogeneity			-0.040	(-0.071 , -0.009)	-0.071	(-0.101 , -0.042)

PLANTS

	1960s		1990s		2010s	
	Mean	95% CI	Mean	95% CI	Mean	95% CI
Intercept	-3.277	(-3.283 , -3.271)	-3.285	(-3.290 , -3.279)	-3.278	(-3.284 , -3.272)
Semi-natural grasslands	0.071	(0.056 , 0.086)	0.144	(0.130 , 0.159)	0.164	(0.150 , 0.178)
Arable	-0.013	(-0.023 , -0.002)			0.018	(0.004 , 0.032)
Improved grasslands			-0.052	(-0.066 , -0.038)	-0.050	(-0.065 , -0.036)
Forest	-0.075	(-0.086 , -0.064)	-0.053	(-0.062 , -0.043)	-0.060	(-0.071 , -0.049)
Urban	0.020	(0.008 , 0.033)	0.021	(0.010 , 0.031)	0.021	(0.010 , 0.032)
Temperature	0.041	(0.021 , 0.061)	0.021	(0.005 , 0.036)	0.043	(0.025 , 0.061)
Precipitation	0.055	(0.032 , 0.078)	0.034	(0.015 , 0.053)	0.038	(0.016 , 0.059)
Microclimate heterogeneity			-0.017	(-0.031 , -0.004)	-0.015	(-0.029 , -0.001)

Positive association   Negative association   No association

Supplementary Table 4. Fixed effects (95% credible interval) for the changes of species richness, beta diversity and community temperature index for three different taxon communities in Great Britain over the long- (from 1960s to 2010s) and short-term (from 1990s to 2010s), based on model eq. 1, 2 and 3 (Methods).

		BIRDS				BUTTERFLIES				PLANTS			
		Long-term		Short-term		Long-term		Short-term		Long-term		Short-term	
		Mean	95% CI	Mean	95% CI	Mean	95% CI	Mean	95% CI	Mean	95% CI	Mean	95% CI
Species richness	Intercept	-5.414	-5.933 , -4.899)	-4.475	(-4.911 , -4.042)	3.218	(3.083 , 3.353)	3.567	(3.469 , 3.664)	7.002	(6.893 , 7.110)	7.263	(7.180 ,7.346)
	Factor time (2010s)	0,019	(0.013 , 0.025)	0,008	(0.002 , 0.014)	0.226	(0.176 ,0.276)	0.047	(0.018 , 0.076)	0,043	(0.031 , 0.055)	0,024	(0.017 , 0.031)
Beta diversity	Intercept	1,687	(1.549 , 1.824)	1,317	(1.201 , 1.433)	0,190	(0.153 , 0.226)	-0,068	(-0.119 , 0.121)	-0,676	(-0.710 , -0.641)	-0,733	(-0.764 , -0.703)
	Factor time (2010s)	0,076	(0.066 , 0.087)	-0,121	(-0.131 , -0.110)	-1,233	(-1.306 , -1.159)	-0,879	-0.951 , -0.808)	-0,148	(-0.166 , -0.131)	-0,042	(-0.053 , -0.031)
Community temperature index	Intercept	2,460	(2.459 , 2.461)	2,453	(2.452 , 2.454)	2,203	(2.202 , 2.204)	2,206	(2.205 , 2.206)	2,099	(2.098 , 2.100)	2,100	(2.099 , 2.101)
	Factor time (2010s)	0,004	(0.002 , 0.006)	0,011	(0.009 , 0.012)	0,002	(0.001 , 0.003)	-0,002	-0.004 , -0.001)	0,009	(0.007 , 0.010)	0,008	(0.007 , 0.009)

Positive association   Negative association   No association

Supplementary Table 5. Comparison of predicted values of changes in species richness (SR), beta diversity (BD) and community temperature index (CTI) using different model structures. Comparisons are between predicted change from models 1-3 (i.e. modelled values from later time period minus earlier time period) and predicted change from versions of model 4 in which only spatial and observer effort controls were included. Sign agreement is the proportion of grid squares for which the sign (positive or negative) were identical, Pearson correlation (two-sided) is the correlation coefficient (all coefficients significant to p<0.001). Average difference is the difference in predicted values across all grid squares in native units (SR: number of species; BD: beta diversity index; CTI: °C), while relative difference is the average difference divided by the range in predicted values across grid cells.

	**Long term**			**Short term**		
	**SR**	**BD**	**CTI**	**SR**	**BD**	**CTI**
**Birds**						
n cells	*2669*	*2670*	*2670*	*2669*	*2670*	*2670*
Sign agreement	0.677	0.936	0.719	0.635	0.963	0.989
Pearson correlation	0.585	0.979	0.268	0.624	0.977	-0.379
Average difference	-0.002	-0.002	-0.001	-0.002	-0.002	-0.002
Relative difference	0	-0.005	-0.001	0	-0.003	-0.003
**Butterflies**						
n cells	*2013*	*2013*	*996*	*2022*	*2022*	*1222*
Sign agreement	1	1	0.71	0.945	0.954	0.791
Pearson correlation	0.73	0.717	0.501	0.519	0.599	0.699
Average difference	-0.68	-0.025	0	-0.562	-0.024	0
Relative difference	-0.027	-0.05	-0.001	-0.018	-0.029	-0.001
**Plants**						
n cells	*2666*	*2666*	*2351*	*2666*	*2666*	*2406*
Sign agreement	0.861	0.981	0.865	0.7	0.941	0.905
Pearson correlation	0.769	0.951	0.469	0.695	0.985	0.238
Average difference	-6.379	0.001	0.001	-3.069	0	-0.001
Relative difference	-0.019	0.006	0.001	-0.011	0.001	-0.001

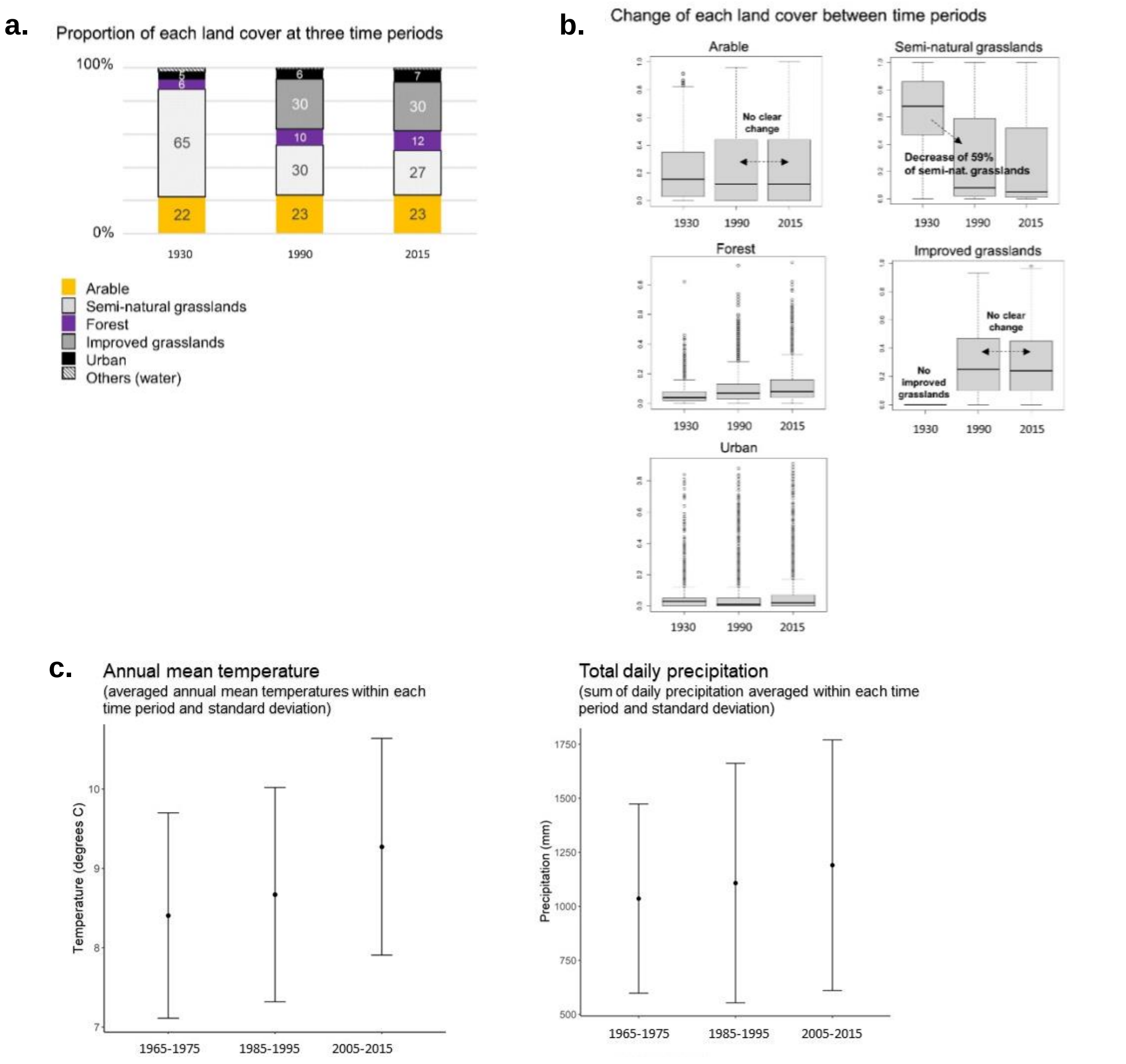

Supplementary Fig. 1 – Land use and climate in Great Britain at three different time periods. a, Proportion of land-use categories across 2670 10 × 10 km grid cells; **b,** Change of each land-use category, where boxes show median (centre line) and interquartile range, with whiskers indicating range excluding outliers (defined as 1.5 × interquartile range from the upper and lower quartiles); and **c,** Averaged annual mean temperature and sum of daily precipitation, standard deviations, and average increase of temperature and precipitation between time periods.

Supplementary Fig. 2 – Consistency of the change models estimating fixed effects. Posterior mean and 95% credible intervals (CI) for the estimated fixed parameters. The circles show the mean fixed effect estimated by the long- and short-term change models of species richness, beta diversity (as a measure of biotic homogenization) and community temperature index, of three different taxa in Great Britain – based on model eq. 4, with solid lines indicating the 95% CI. The triangles show the mean fixed effect derived from the 10-fold cross-validation analysis on the same models, with dotted lines indicating the 95% CI. Labels' abbreviations: SN = semi-natural grasslands; AR = arable; FR = forest; UR = urban; IG = improved grasslands; MicroClim = microclimate heterogeneity. Number of grid cells analysed as follows: Birds 2670 across all analyses; Butterflies species richness and beta diversity: 2013 long term and 2022 short term, CTI 996 long term and 1222 short term; Plants species richness and beta diversity 2666 at both long and short term, CTI 2351 long term and 2406 short term. Figure extends over six pages: long and short term change for birds, butterflies and plants.

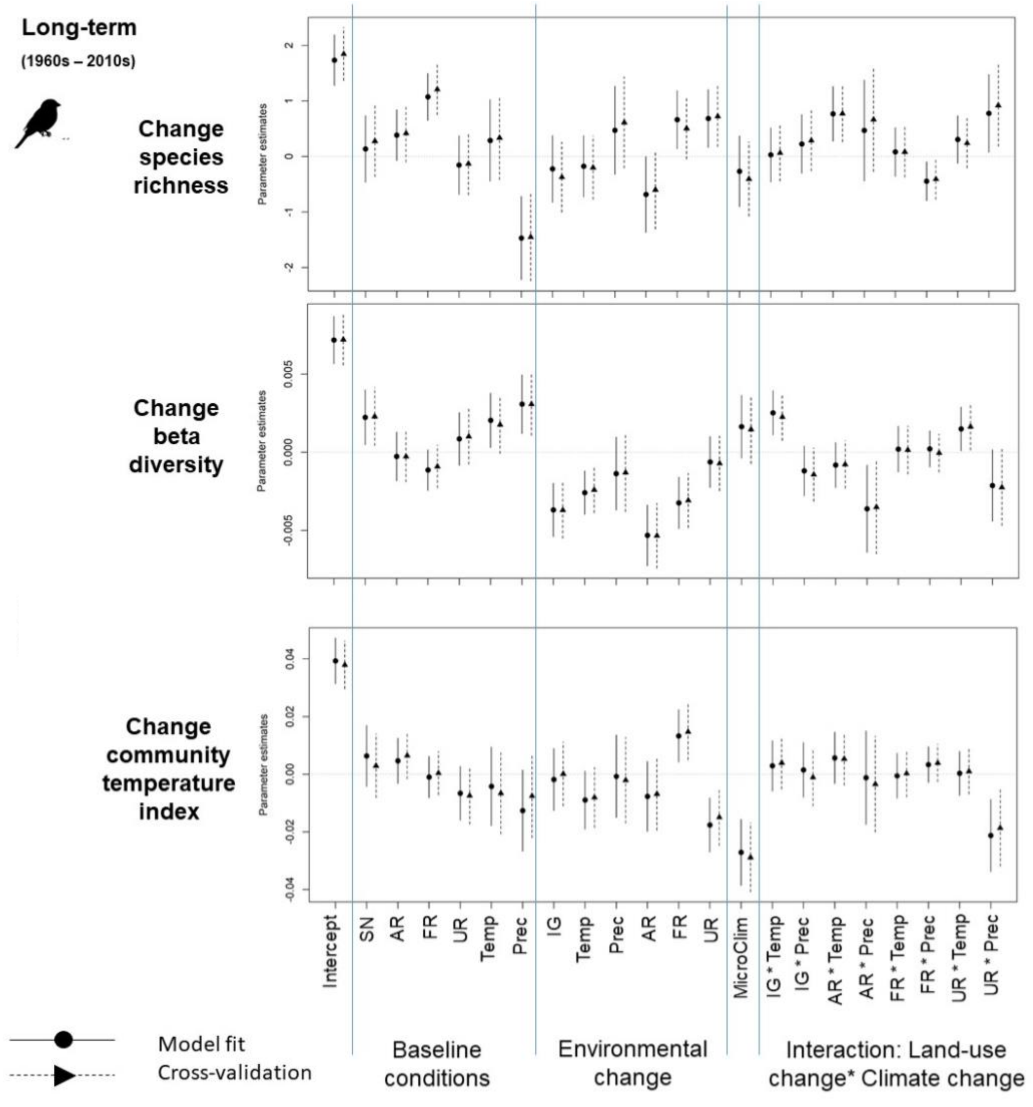

Supplementary Fig. 2 continued.

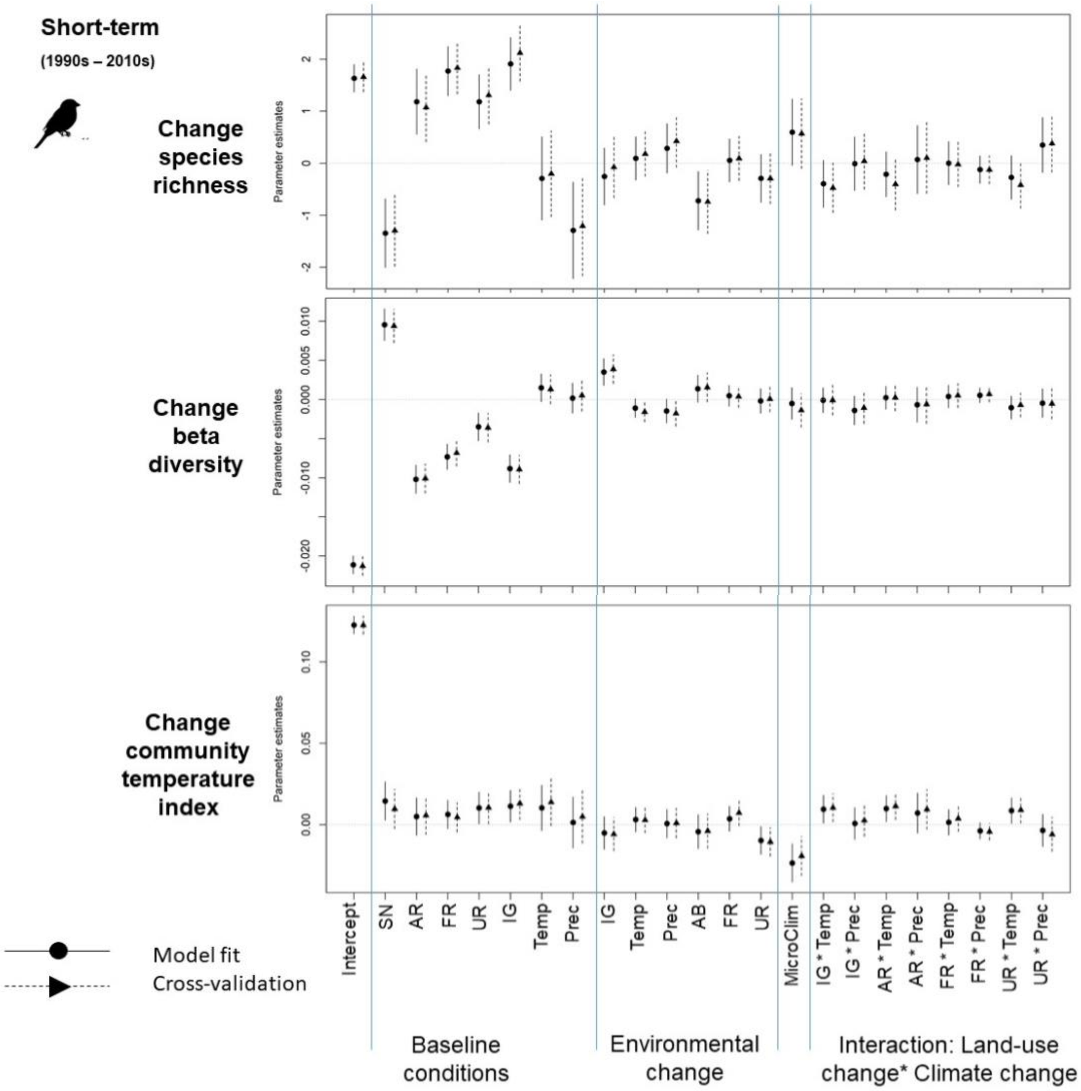

Supplementary Fig. 2 continued.

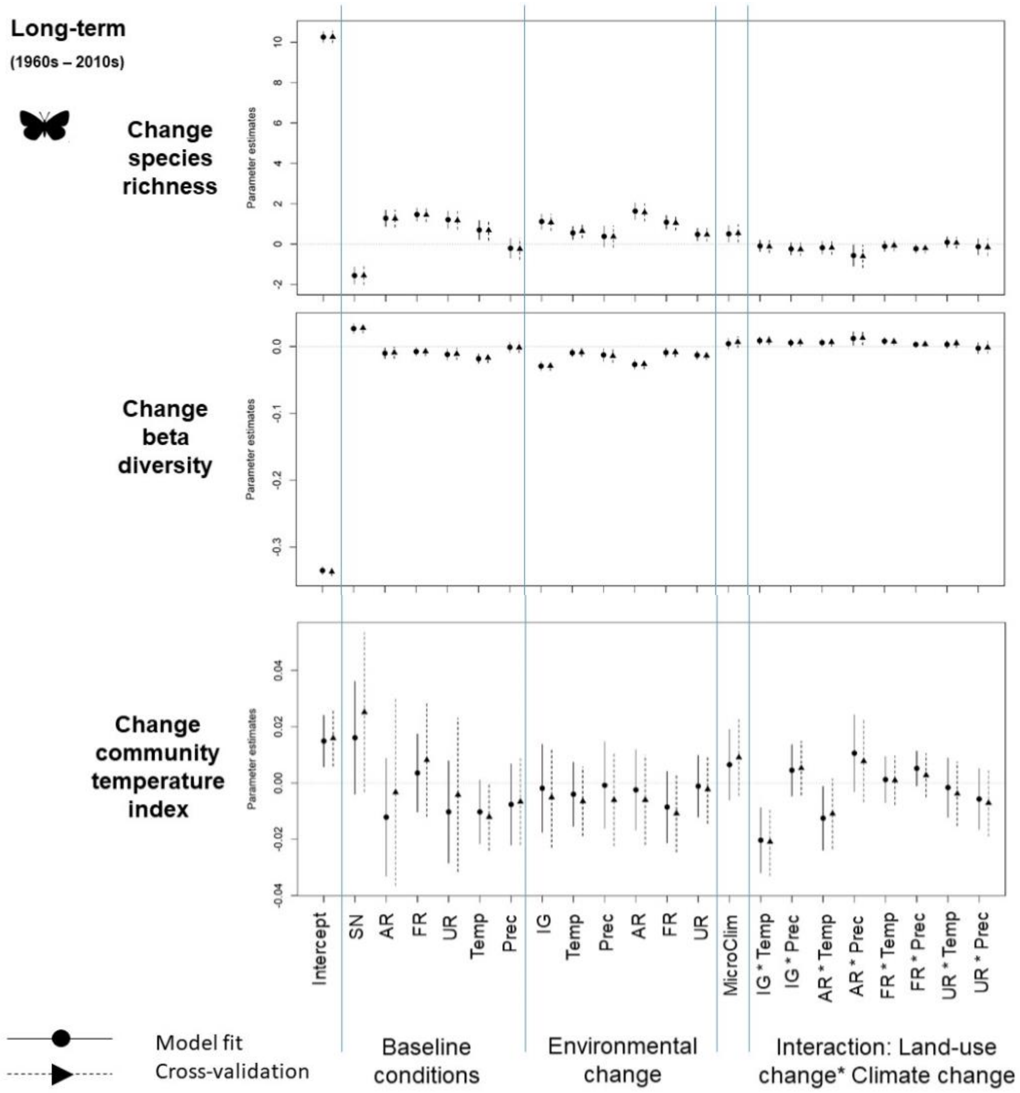

Supplementary Fig. 2 continued.

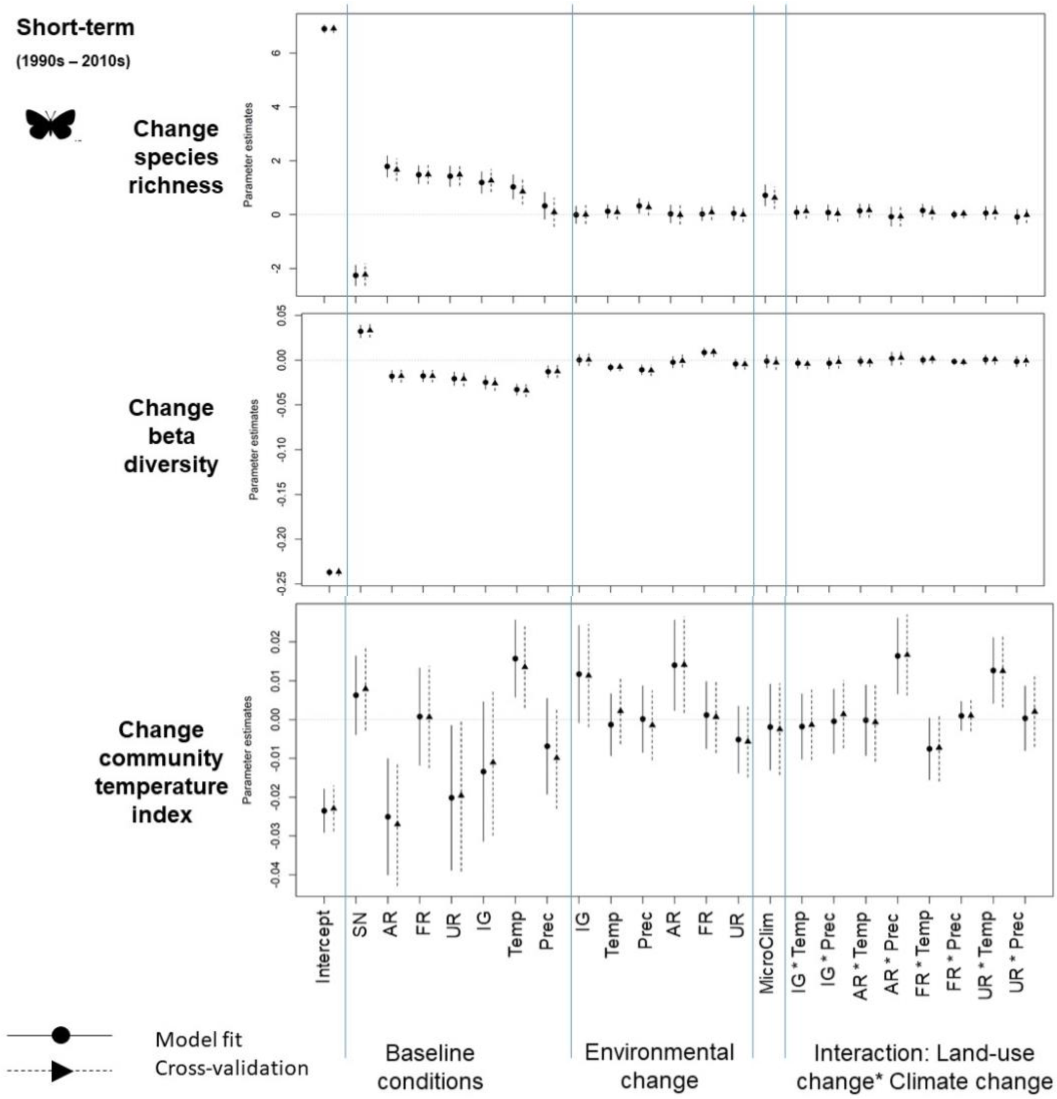

Supplementary Fig. 2 continued.

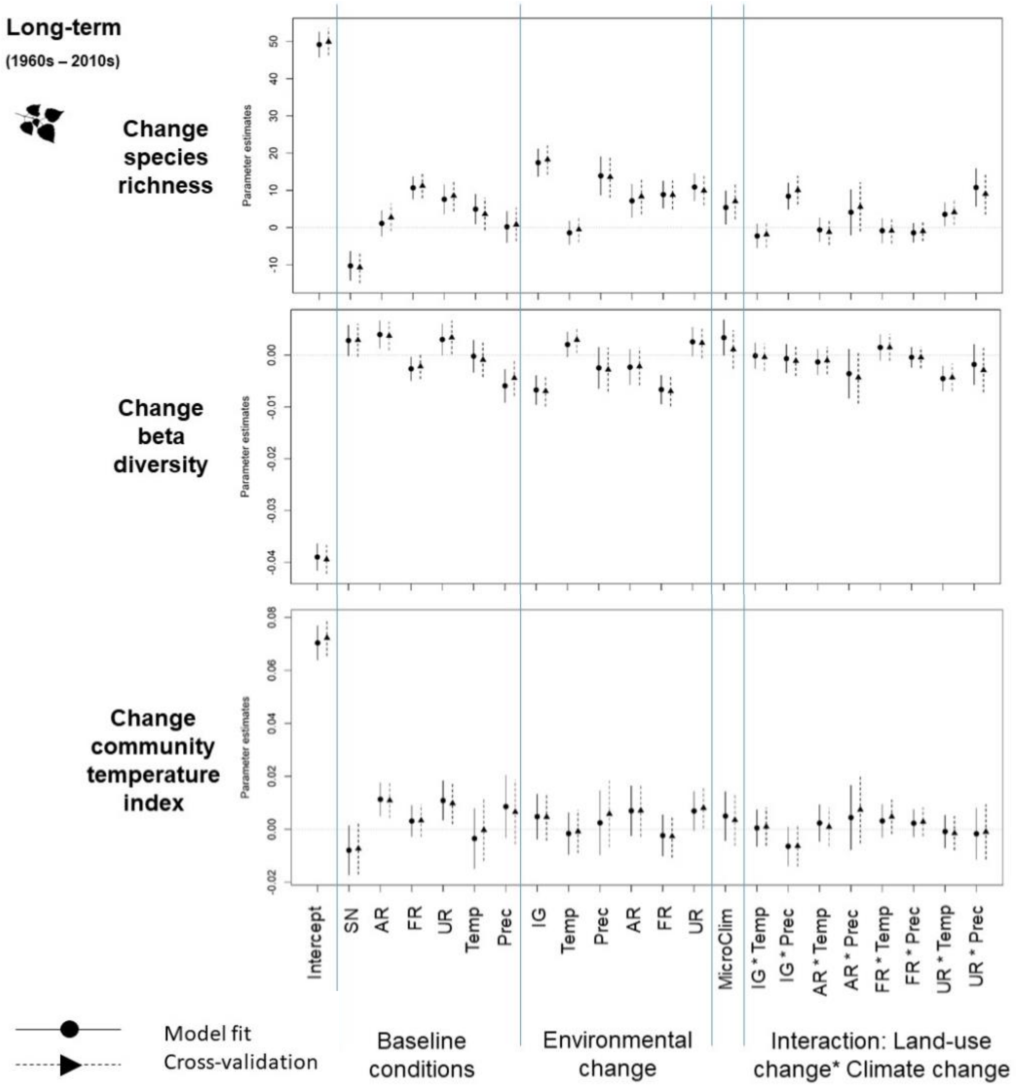

Supplementary Fig. 2 continued.

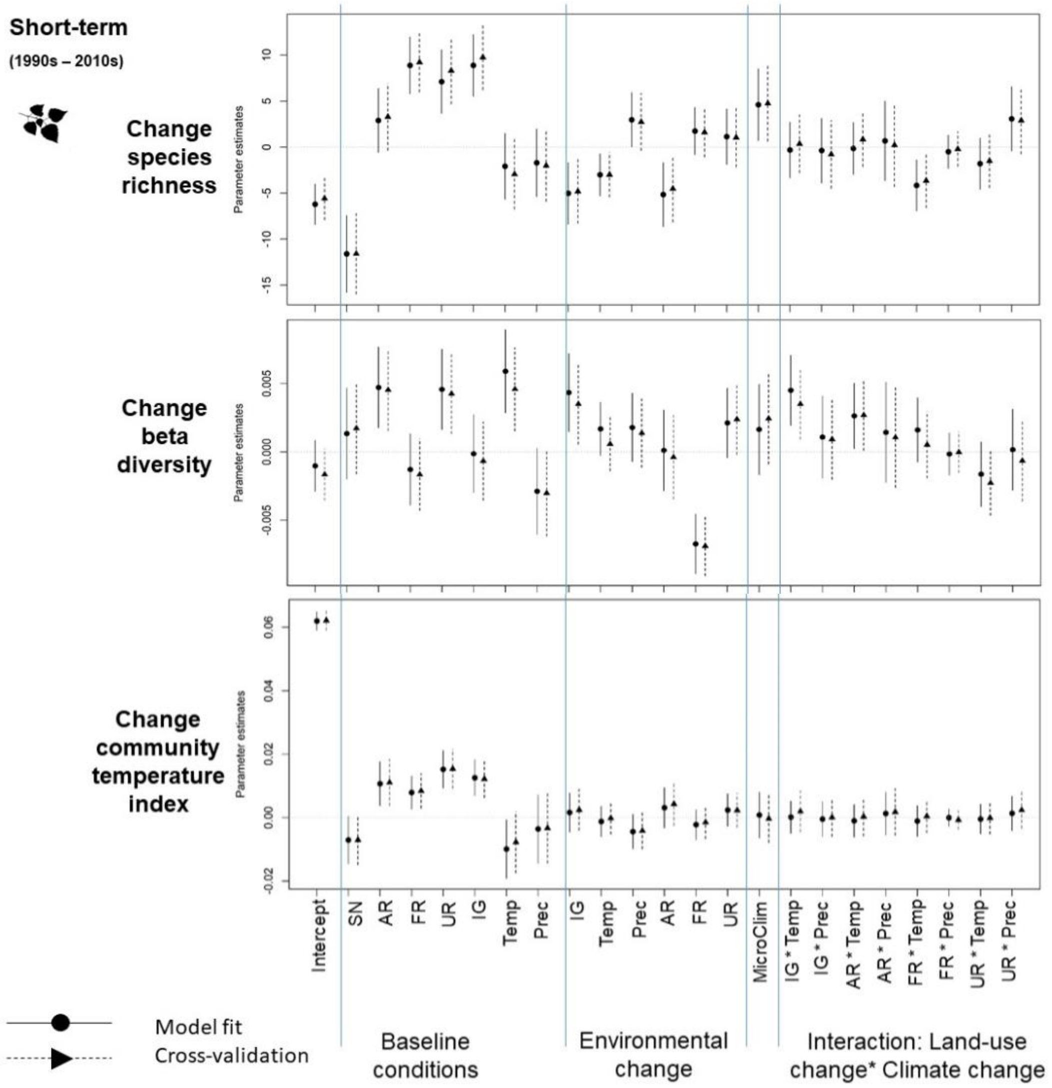

Supplementary Fig. 3 – Associations between interacting effects of land-use and climate change and biodiversity change. The interaction plots show the predictions of the interaction terms (i.e., effect of "*land-use change + interaction*") with a shadow purple gradient for positive interactions, and a shadow orange gradient for negative interactions, considering two scenarios: a constant increase and decrease in temperature of +0.5ºC, -0.5 ºC; or a constant increase and decrease of precipitation of +500mm -500mm across all grid-cells (depending if the interaction was found between land-use and temperature change, or land-use and precipitation change). Only significant interactions (with the 95% posterior distribution of the estimated mean of the coefficient not including zero) are shown (Figure 3). The x-axis refers to the change in land use. The solid black line is the mean effect of the land-use change without considering the interaction (we used a dashed black line if no association between change of land use and biodiversity was found; Figure 3). Interpretability: parallel shadows would indicate no interaction effect while crossing slopes support the presence of an interaction. Number of grid cells analysed as follows: Birds 2670 across all analyses; Butterflies species richness and beta diversity: 2013 long term and 2022 short term, CTI 996 long term and 1222 short term; Plants species richness and beta diversity 2666 at both long and short term, CTI 2351 long term and 2406 short term. Figure extends over three pages: birds, butterflies and plants.

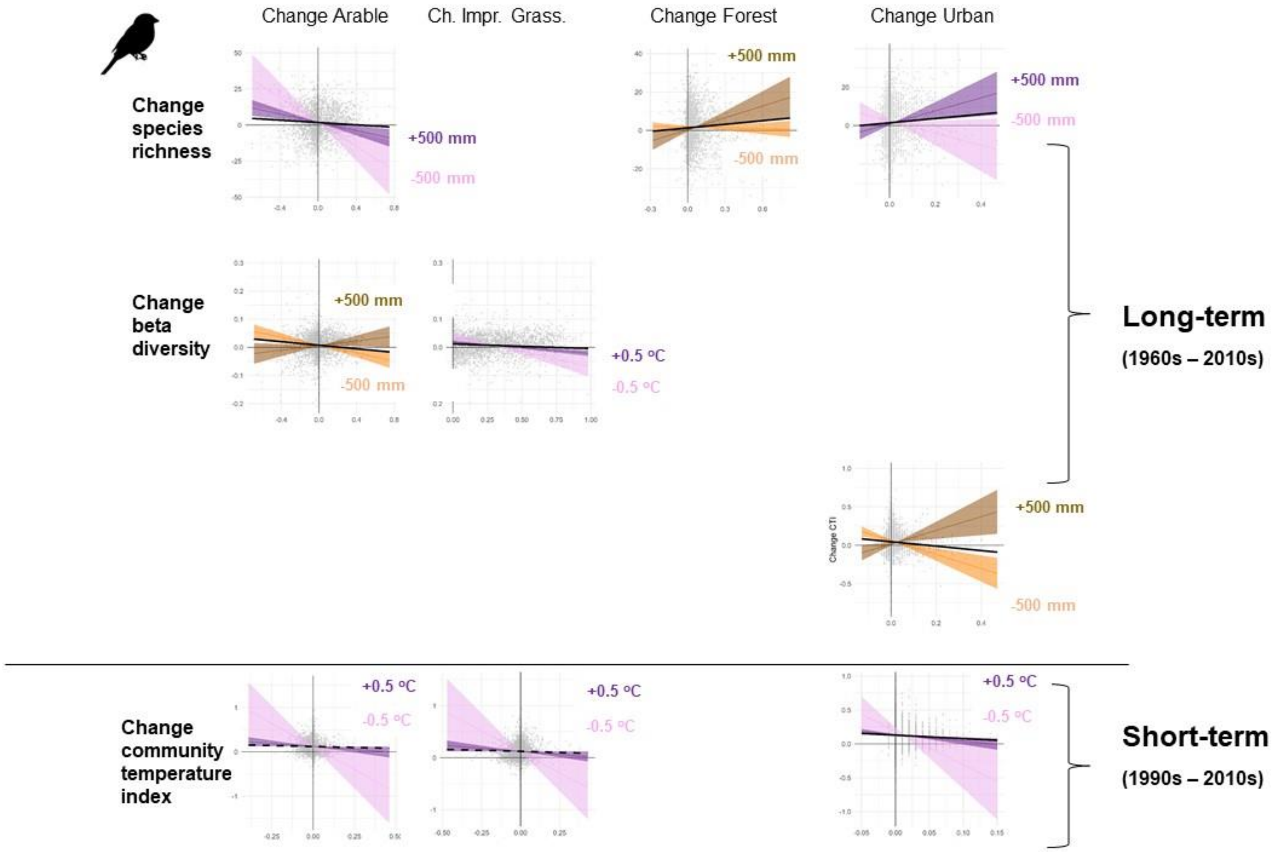

Supplementary Fig. 3 continued.

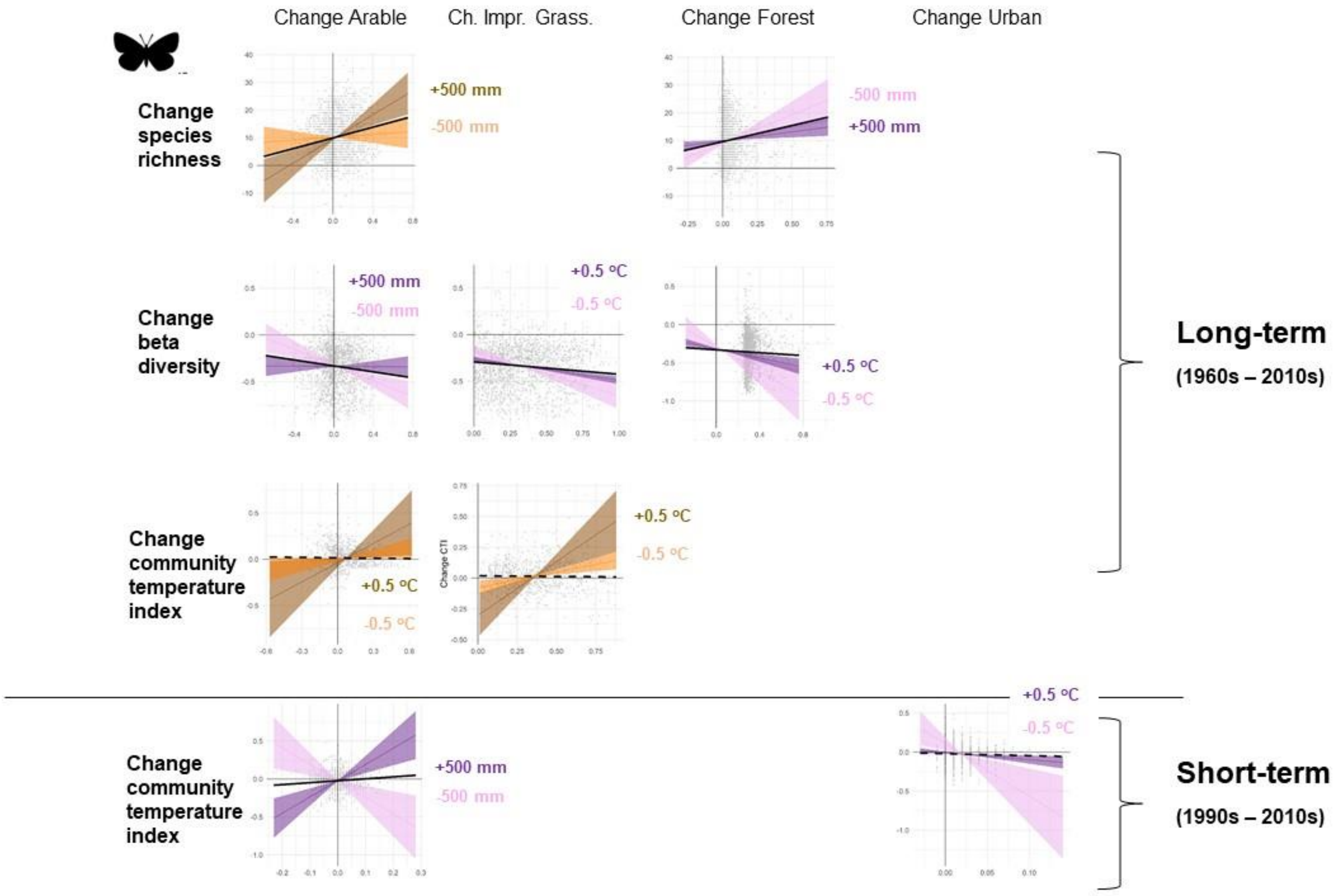

Supplementary Fig. 3 continued.

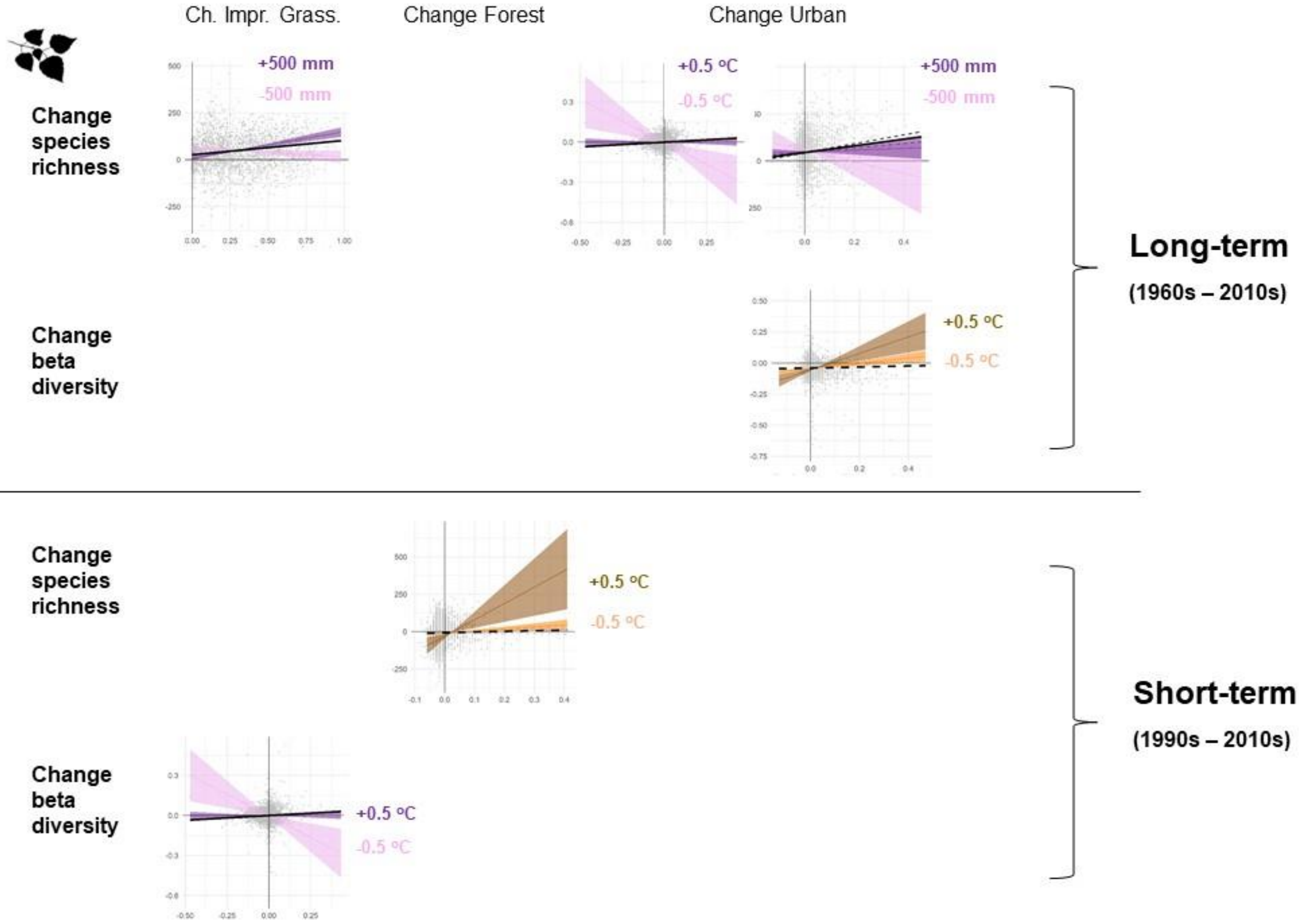

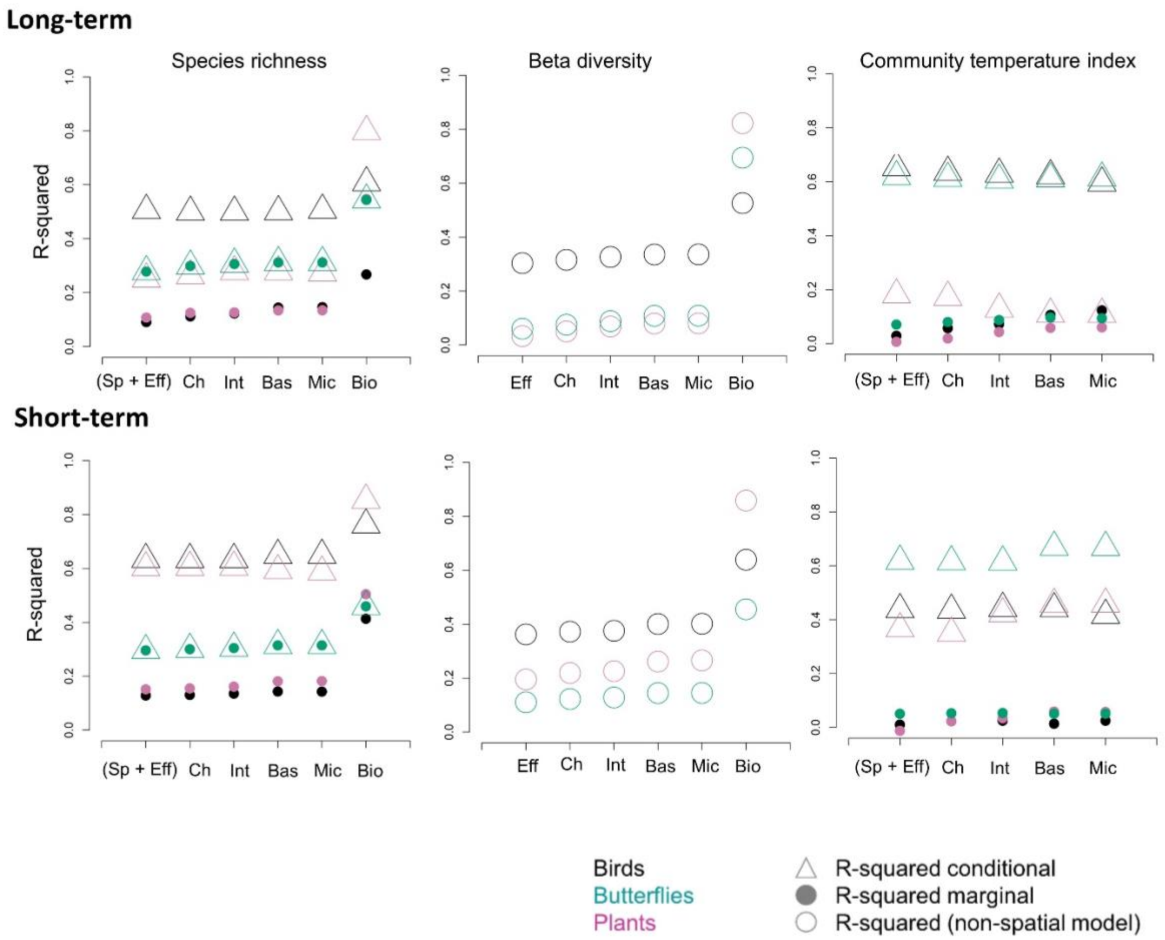

Supplementary Fig. 4 – Comparison of R-square marginal and R-square conditional. The panels show the amount of variation in biodiversity change explained by each one of the explanatory variables of interest, namely changes in climate and land use (Ch), climate-land-use change interactions (Int), baseline conditions of climate and land use (Bas), microclimate heterogeneity (Mic), and baseline biodiversity conditions (Bio) – based on model eq. 4 – for three different taxon communities in Great Britain, over the long (1960s to 2010s) and short temporal scales (1990s to 2010s). The models have been run in consecutive steps, starting with a model that only contained the spatial term, the estimated recorder effort at the beginning of the period of change, and the change of estimated recorder effort, and adding a group of explanatory variables at each step, as defined by the x-axis. R-square was calculated at each step. Note that models for beta diversity do not include spatial dependency and that CTI does not include baseline biodiversity. Number of grid cells analysed as follows: Birds 2670 across all analyses; Butterflies species richness and beta diversity: 2013 long term and 2022 short term, CTI 996 long term and 1222 short term; Plants species richness and beta diversity 2666 at both long and short term, CTI 2351 long term and 2406 short term.

Supplementary Fig. 5 – Consistency of the LCBD models estimating fixed effects. Posterior mean and 95% credible intervals (CI) for the estimated fixed parameters. The circles show the mean fixed effect estimated at each time period (1960s, 1990s and 2010s) for three different taxa in Great Britain – based on model eq. 5, with solid lines indicating the 95% CI. The triangles show the mean fixed effect derived from the 10-fold cross-validation analysis on the same models, with dotted lines indicating the 95% CI. Figure continues onto next page.

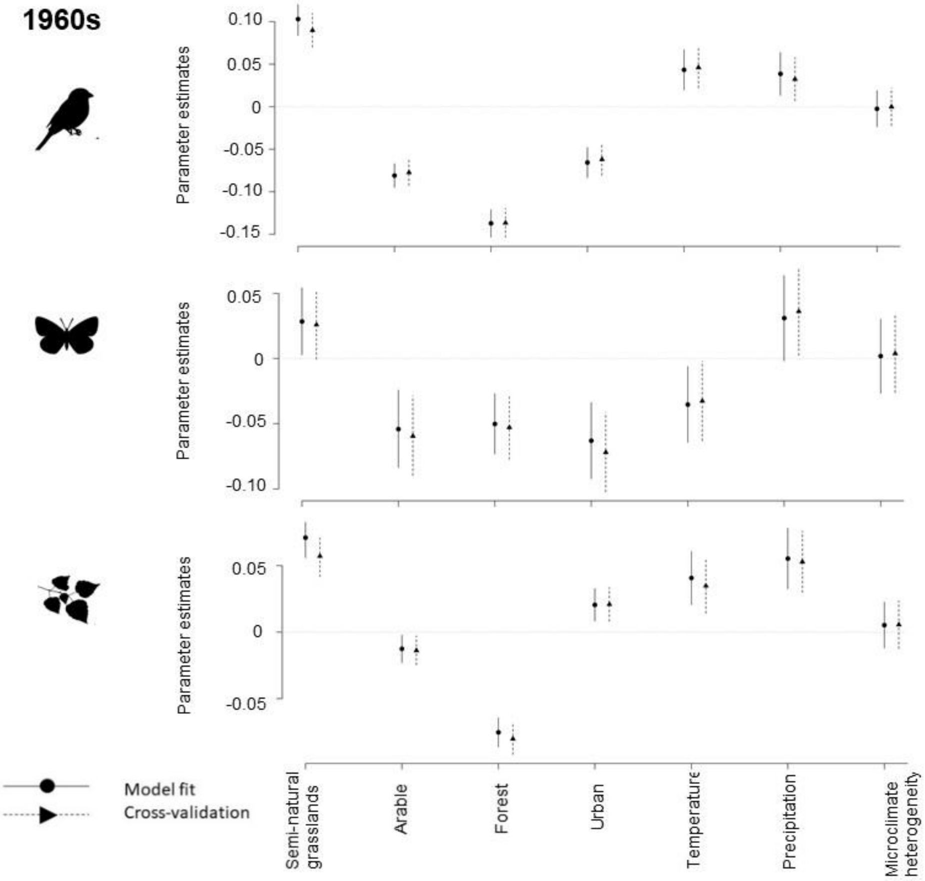

Supplementary Fig. 5 continued.

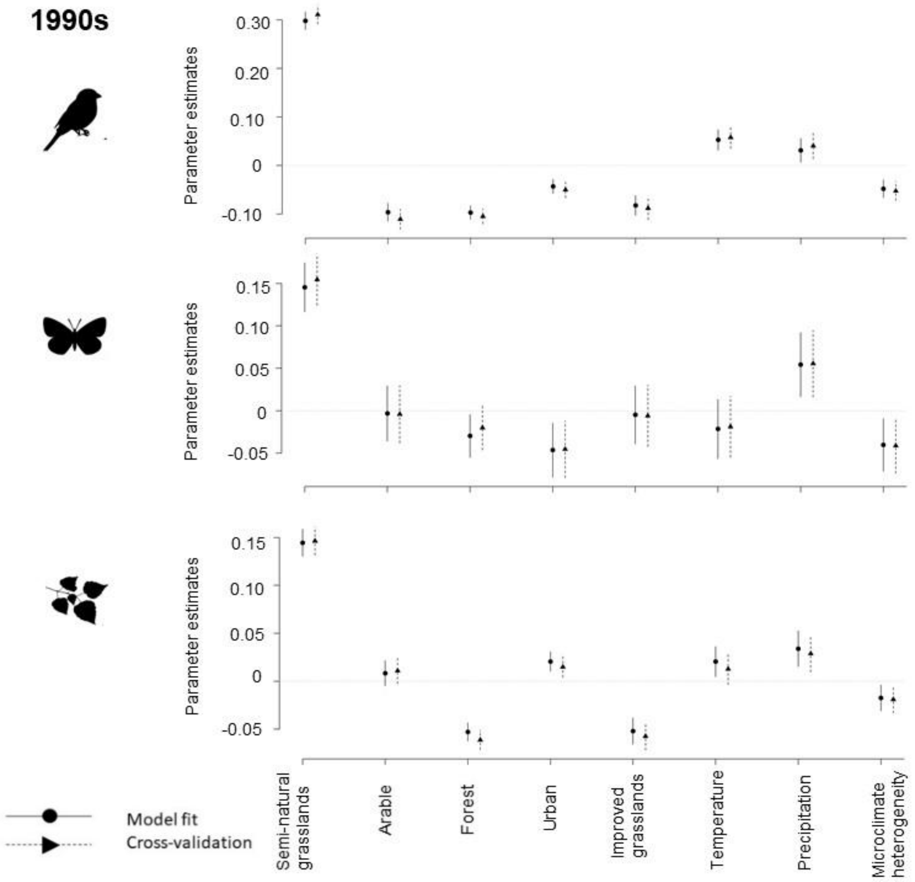

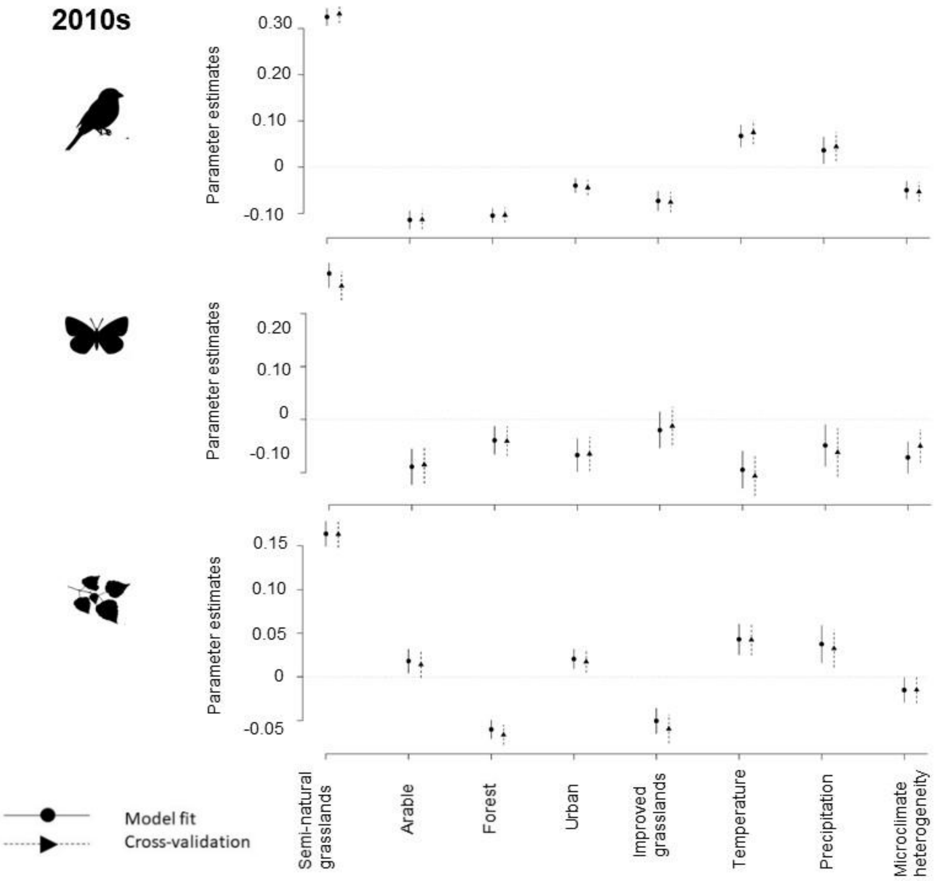
2010s
Parameter estimates
0.30
0.20
0.10
0
-0.10
Parameter estimates
0.20
0.10
0
-0.10
Parameter estimates
0.15
0.10
0.05
0
-0.05
Semi-natural grasslands
Arable
Forest
Urban
Improved grasslands
Temperature
Precipitation
Microclimate heterogeneity
Model fit
Cross-validation

a.

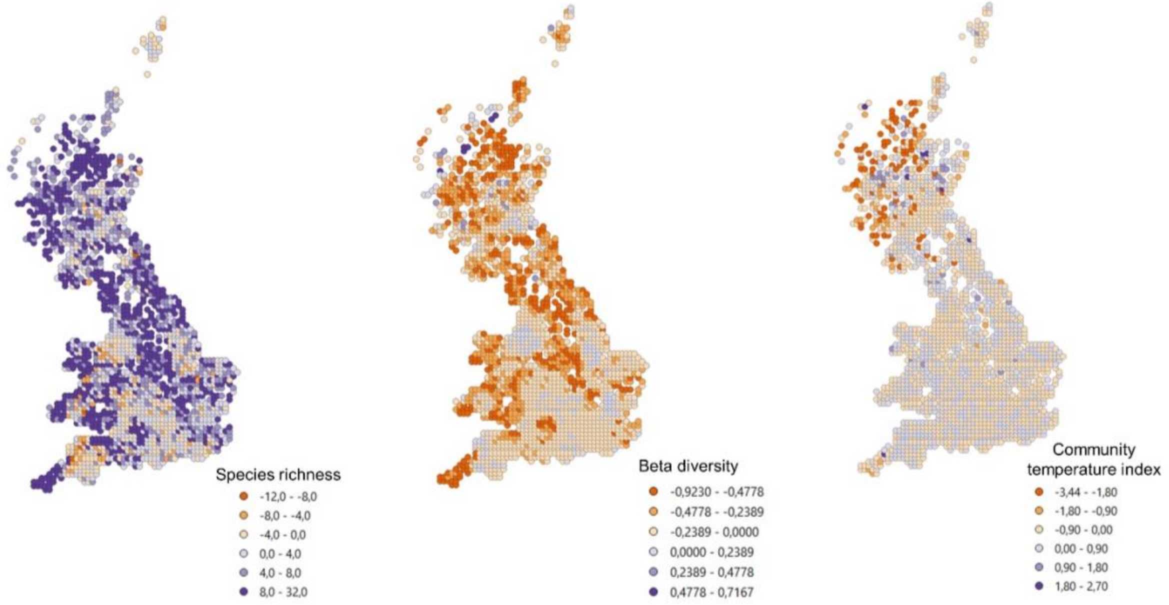

b.

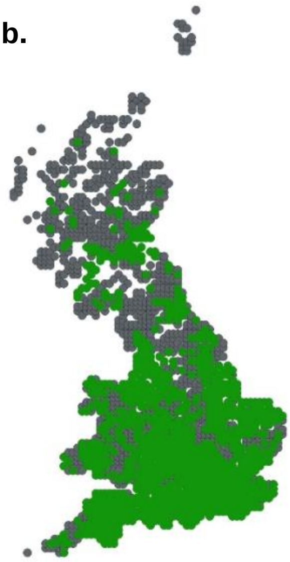

Supplementary Fig. 6 – Short-term biodiversity change for the British butterflies. a, Maps showing change in species richness, beta diversity (as a measure of biotic homogenization) and CTI at the short-term (from the 1990s to 2010s). **b,** Map showing the grid cells with observations recorded for the short-term period (dark grey) and the grid cells eligible for the biodiversity change analysis (i.e., above the 10 species richness cut off, in green).

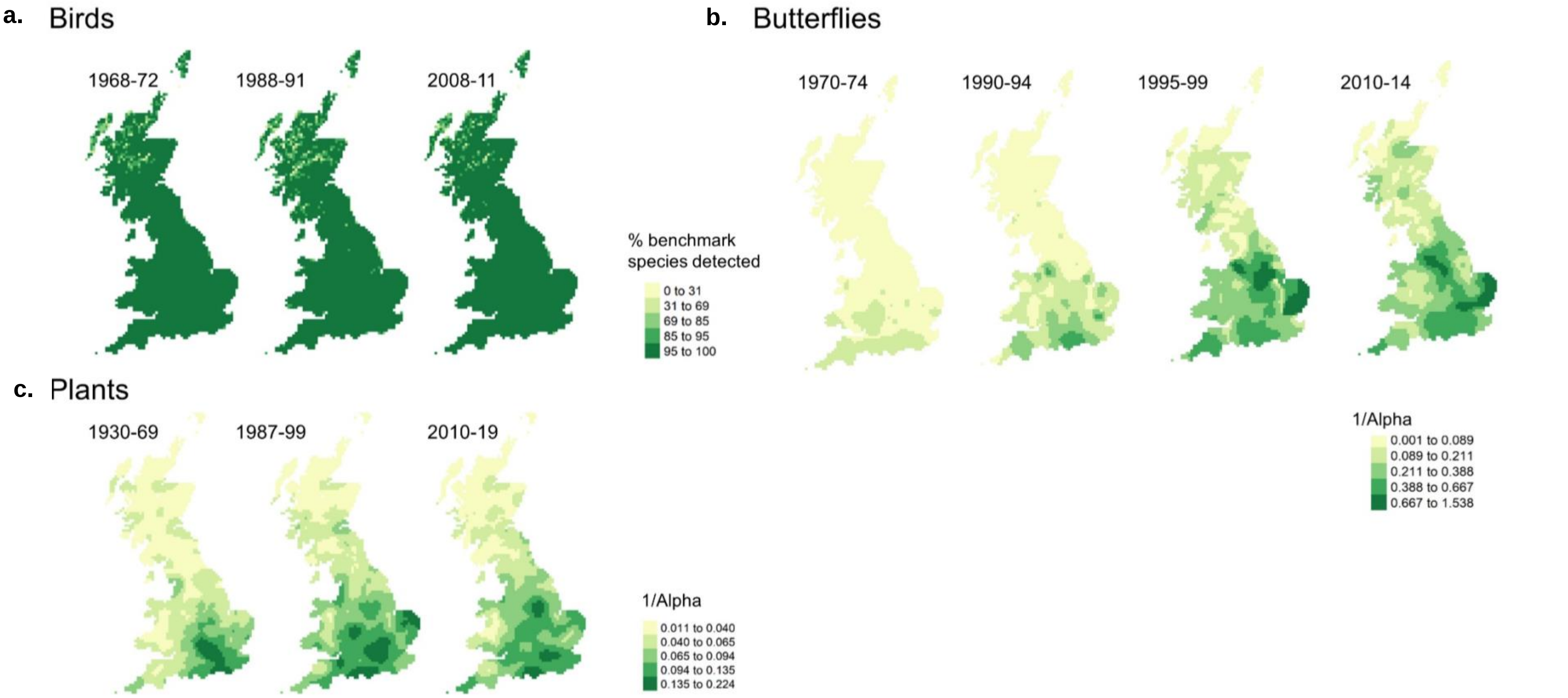

Supplementary Fig. 7 – Estimated recorder effort for the British breeding birds, British butterflies, and British plants atlases at different time periods. a, b, c, Maps represent the estimated recorder effort obtained using the method Frescalo (Hill 2012), as percentage of benchmark species detected (in birds) and as the invers of Alpha - sampling effort multiplier (in butterflies and plants) as described in Methods. A higher percentage of species and higher inverse of Alpha indicate higher recorder effort.

Supplementary Fig. 8 – Associations between change in biodiversity and change in and baseline of environmental variables. Relationships between changes in Species richness (***a***), Beta diversity (***b***) and Community temperature index (***c***) and changes in land use and climate for three taxa in Great Britain, based on model eq. 4 (Methods). Solid lines represent the mean effect of each environmental variable over the change in biodiversity and dashed lines show the 95% credible interval. Grey dots represent the observed values. Notice that only the associations with a 95% credible interval within the positive or negative range are shown. Number of grid cells analysed as follows: Birds 2670 across all analyses; Butterflies species richness and beta diversity: 2013 long term and 2022 short term, CTI 996 long term and 1222 short term; Plants species richness and beta diversity 2666 at both long and short term, CTI 2351 long term and 2406 short term.

a. Species richness

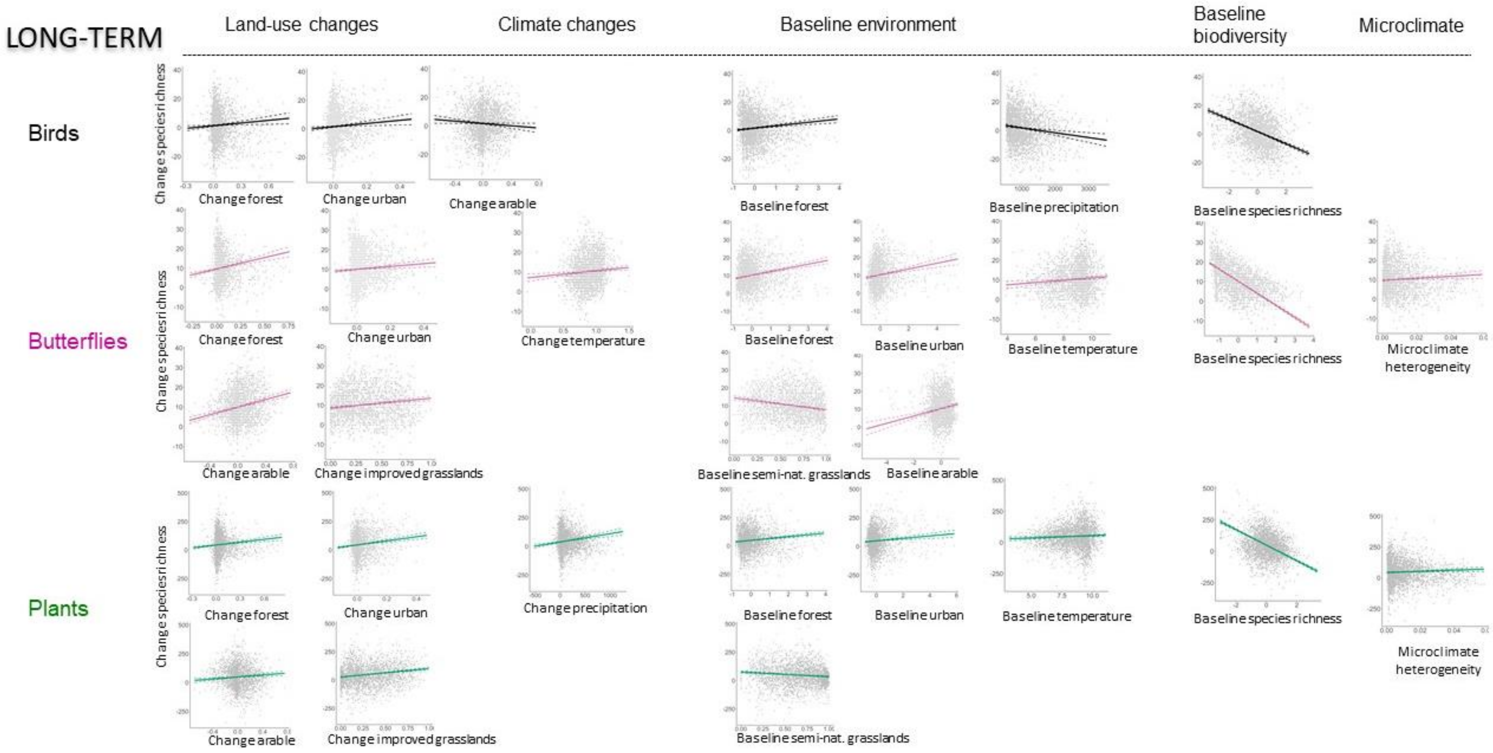

Supplementary Fig. 8a continued.

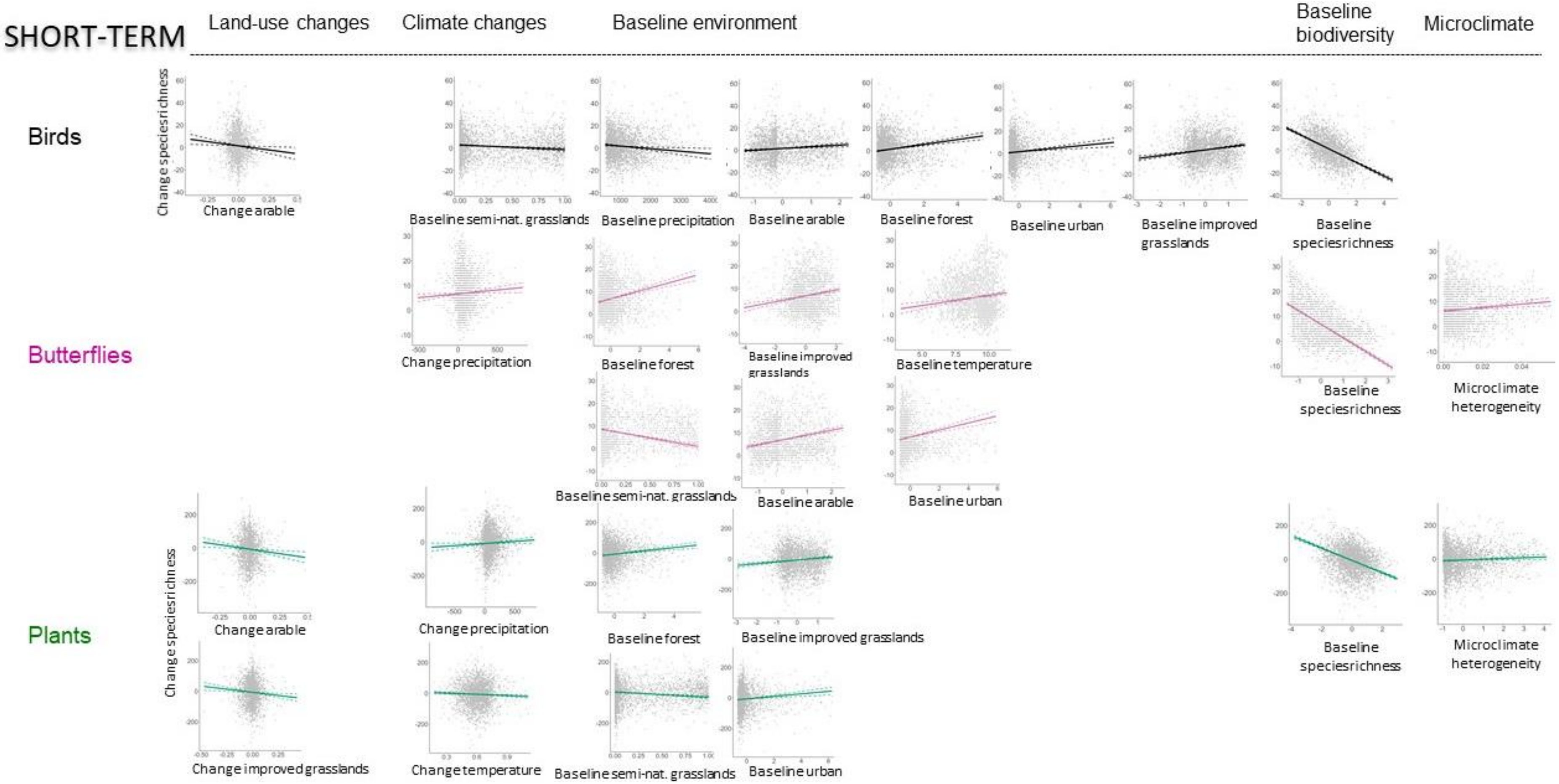

Supplementary Fig 8b. Beta diversity

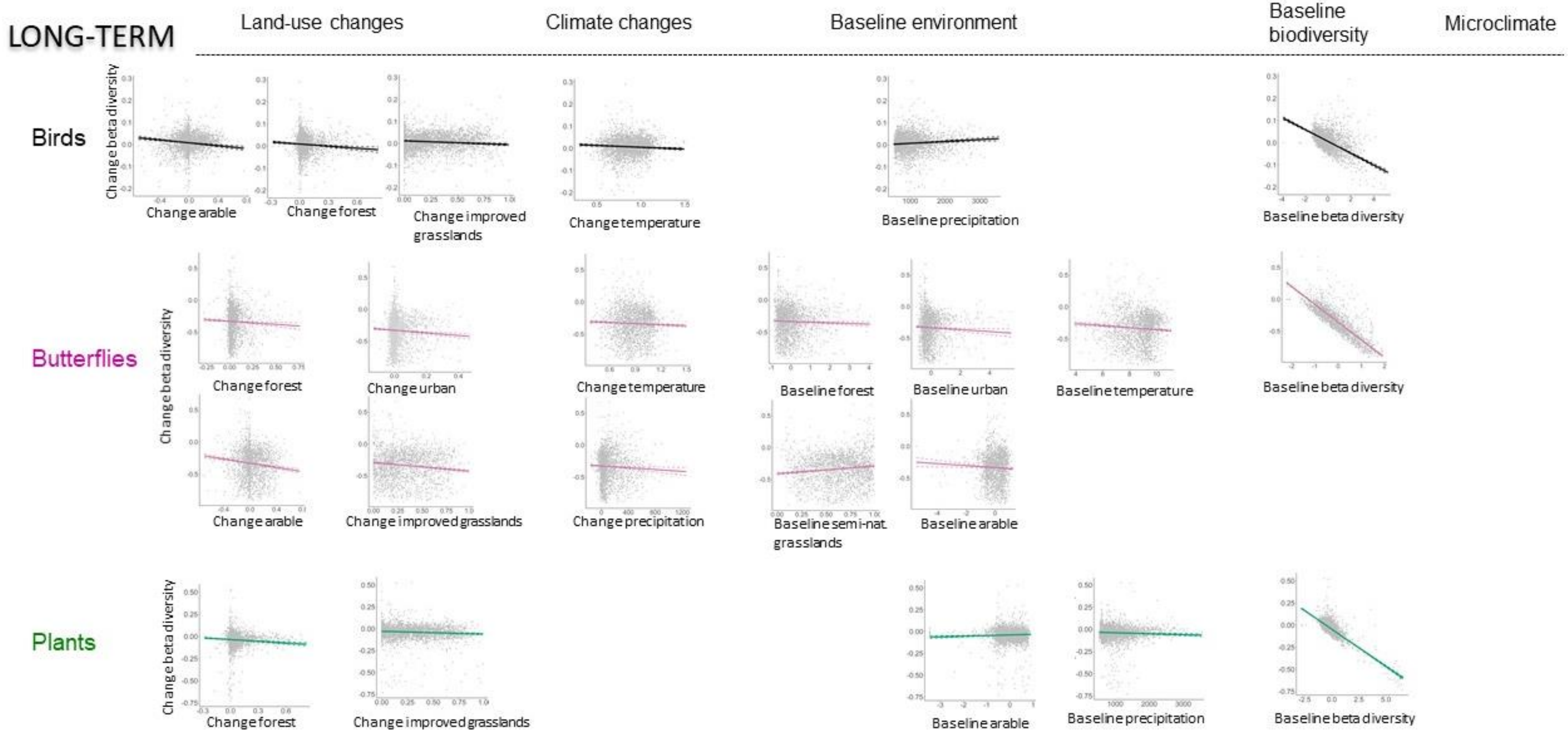

Supplementary Fig. 8b continued.

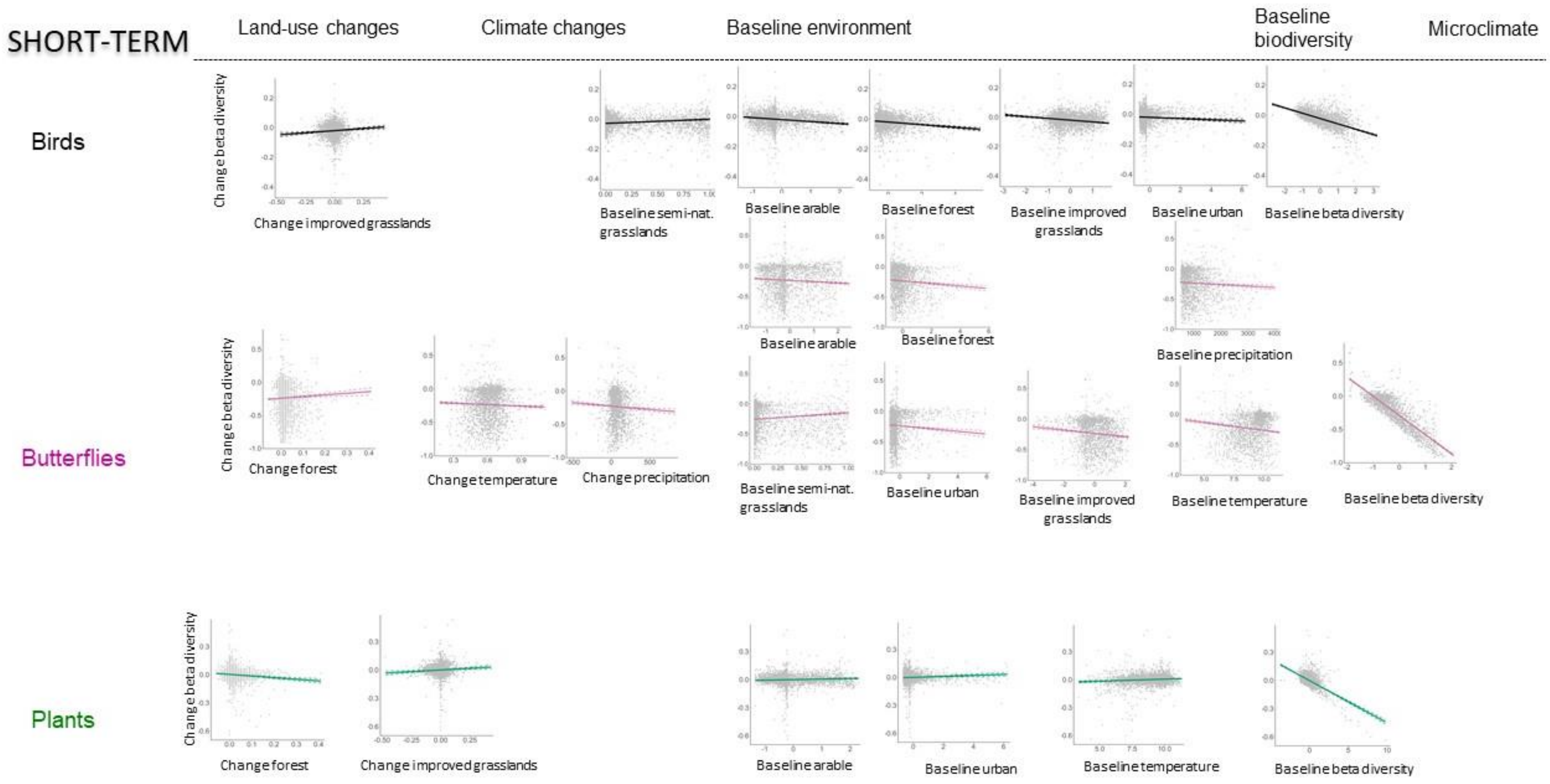

Supplementary Figure 8c. Community Temperature Index

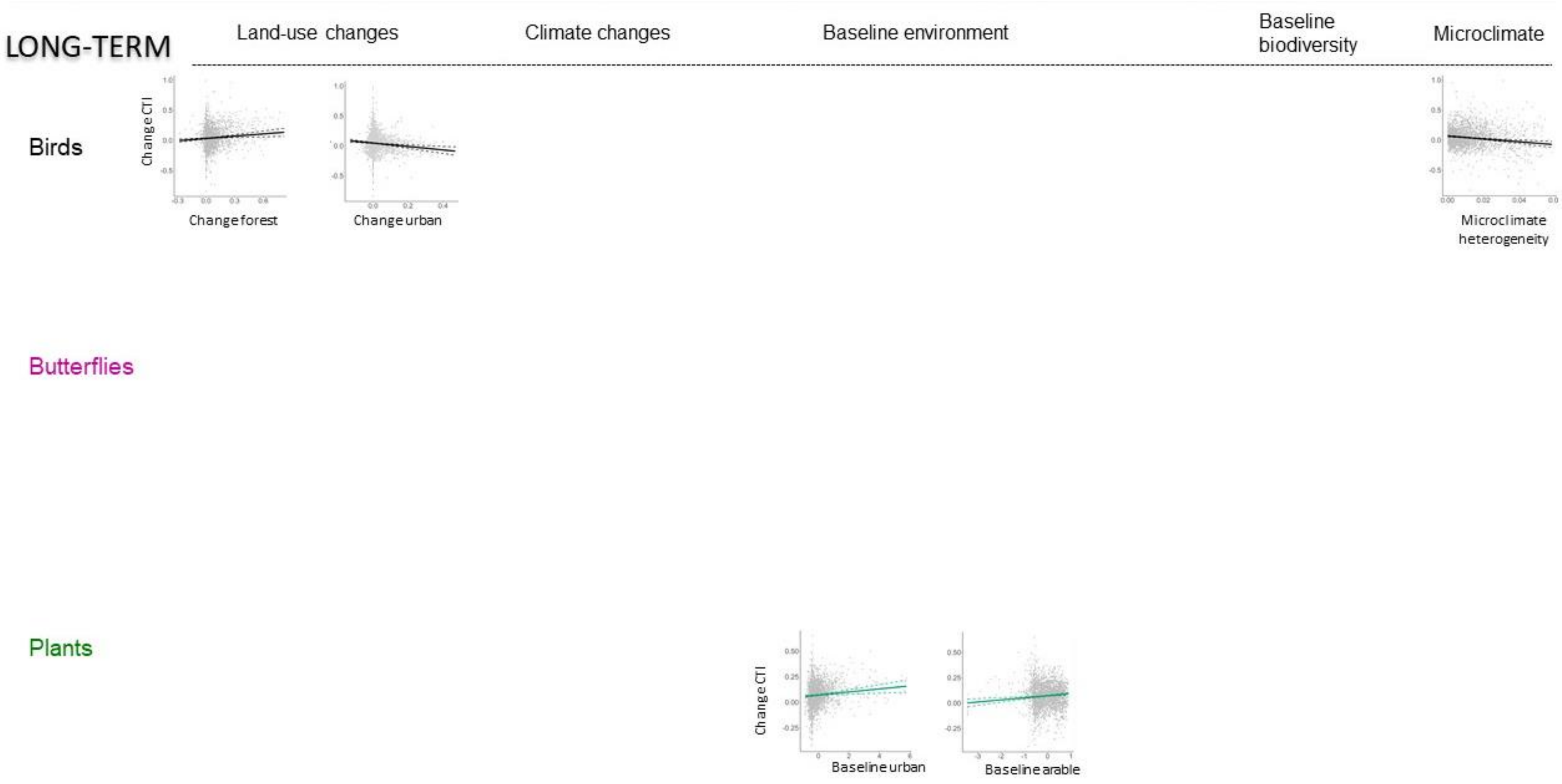

Supplementary Fig. 8c continued.

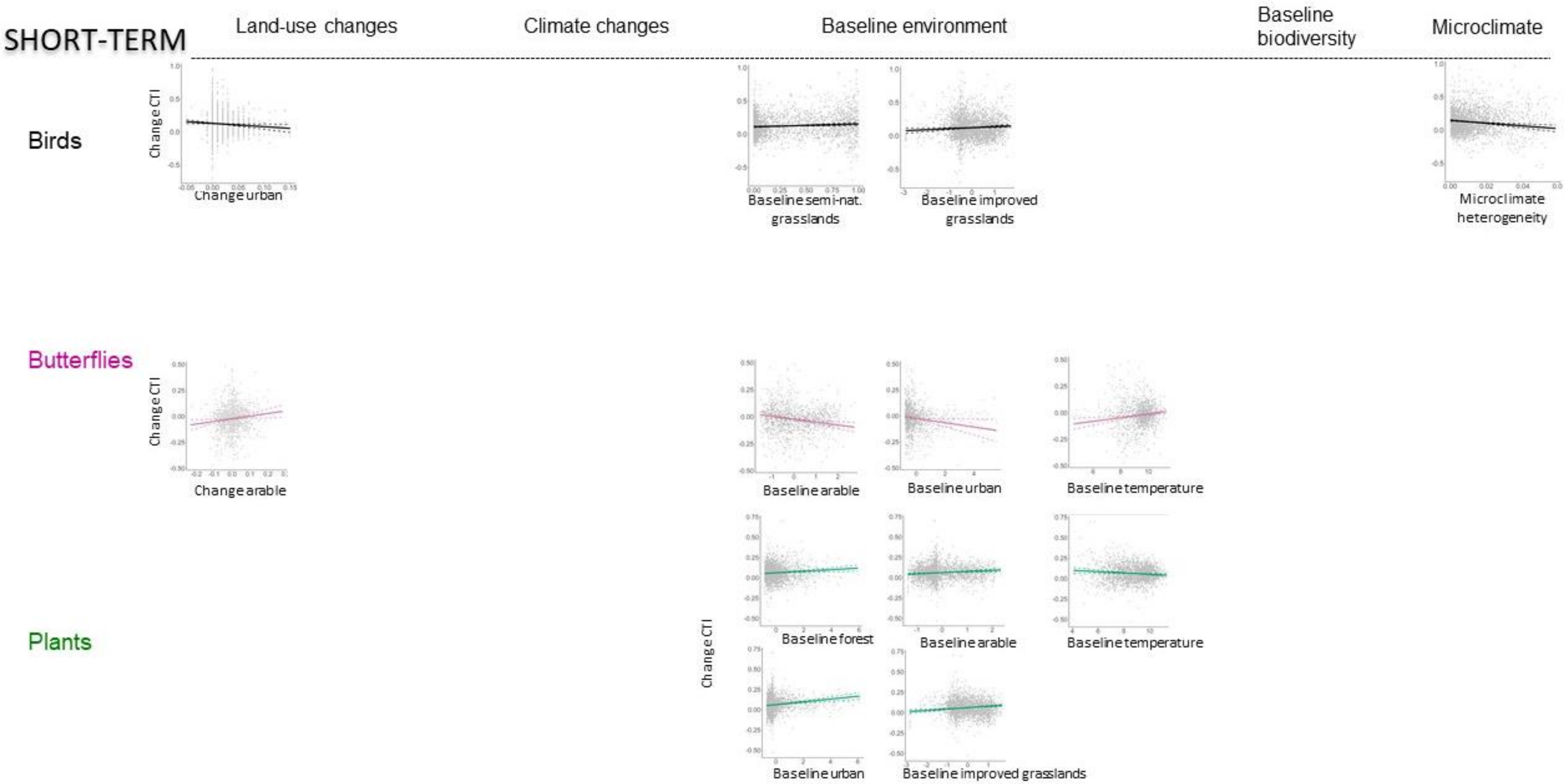

Supplementary Notes

Taxonomic filtering of observation data

From the complete list of species registered in the breeding bird atlases, we first merged common, Scottish and parrot crossbill *Loxia curvirostra, L. scotica* and *L. pytyopsittacus,* as well as common and lesser redpoll *Acanthis flammea* and *A. cabaret*. The three crossbills and the two redpolls have no DNA differences and there are innumerable middle forms[1,2]. Additionally, crossbills and redpolls are hard to separate and therefore prone to identification errors in field. Then, we selected all bird species from Category A (species recorded in an apparently natural state at least once since 1 January 1950, from the official list *https://bou.org.uk/british-list/species-categories/* managed by the British Ornithologist Union Records Committee). This included all Western Palearctic species which, despite being uncommon in Great Britain (hereafter, GB), might have reached by their own means from other Western Palearctic locations (natural colonization). From the complete list of species recorded by the Butterflies for the New Millennium recording scheme, we excluded species that have not been recorded consistently over time; specifically brown argus *Aricia agestis* and northern brown argus *A. artaxerxes* due to taxonomic and field identification issues, and a range of scarce immigrant species. All other species were retained, including the common migratory species clouded yellow *Colias croceus* and painted lady *Vanessa cardui*, as their spatial occurrence can be potentially related to climate and land-use changes in GB. For plants, we included species that could be reliably compared over the three atlas periods[3]. Black poplar *Populus nigra* was removed due to it commonly being used in plantation forestry.

The iCAR model structure [4]

$\gamma_i \mid \gamma_{i-1} \sim \text{Gaussian}\left(\frac{1}{Ni}\sum_{j=1}^{n} aij\,\gamma j,\ S_i^2\right)$

i and *j* identifies each one of the spatial-dependency matrix-structure (rows and columns). N_i is the total number of neighbors of grid *i.* a_{ij} is the element containing the spatial weights, which is 1 if grids *i* and *j* are neighbors or 0 otherwise.; *n* refers to the total number of grids (from 1 to *n*). S_i^2 is the within-

grid variance, defined as: $S_i^2 = \frac{\sigma_\gamma^2}{Ni}$, where σ_γ^2 is the between-grid variance and controls the amount of variation between the spatially-structured random effects.

Dealing with collinearity

The overall goal of this study is to disentangle baseline and interacting effects of land-use and climate change on biodiversity across the 20th century. Hence, presence of collinearity among the explanatory variables and potential cofounding effects is a major concern that needs to be addressed. We found collinearity among: (1) land-use categories, especially between semi-natural grasslands and arable in the 1960s, and semi-natural grasslands, arable, and improved grasslands in the 1990s (Variance Inflation Factor > 13, and > 14, respectively); and (2) changes of land-use categories (VIF ranging from 5.8 to 68.4 for the long-term, and 4.5 to 34.0 for the short-term). We also found a potential for confounding effects in model eq. 4, in terms of using baseline biodiversity (richness and beta diversity) together with estimated recorder effort, due to their levels of correlation.

To reduce collinearity among land-use categories, we used the sequential regression approach[6-8]. In sequential regression, we first need to sort the explanatory variables following an order of importance that follows an ecological reasoning. The first variable will remain as it is. The second will be regressed to the first and the residuals of this regression represent the independent contribution of the second variable after accounting for the effect of the first (and so on)[8]. By using the residuals as new variables in the model, any collinearity among original variables is removed[6,7]. For each time period, we fitted a Beta GLM with logit-link function to regress arable, forest and urban land-cover categories independently against semi-natural grasslands, and to regress improved grasslands against semi-natural grasslands and the residuals of arable. To reduce confounding effects beween changes of land use, we used a slightly different approach. Because we are interested in the interactions between changes of land use and climate, we did not want to transform these variables using sequential regression. Instead, and because the proportion of urban, forest, improved grasslands and arable increased largely at the expense of semi-natural grasslands, we decided to exclude “change of semi-natural grasslands” as explanatory variable from the models. To avoid potential confounding effects when using baseline biodiversity and estimated recorder effort as explanatory variables in the

same model, we used sequential regression[9]. We fitted a Negative Binomial GLM (with natural logarithm link-function) and a Beta GLM (with logit link-function) for each taxon and time period (1960s and 1990s), to regress richness and beta diversity, respectively, against estimated recorder effort at the same time period. The residuals of each one of these regressions were used as the new baseline biodiversity explanatory variable.

After applying sequential regression analysis and removing change in semi-natural grasslands, VIF dropped below 2.4. However, for the butterfly dataset, limited collinearity was still recorded once we corrected for uneven recorder effort using the richness grid-cell cut-off approach (CTI analysis). Specifically, in the long-term analysis, VIF between semi-natural grasslands and the residuals of arable and urban ranged from 5.0 to 6.5, while in the short-term, VIF among the residuals of arable, urban, and improved grasslands ranged from 4.3 to 7.2.

Assessing spatial dependency

To assess the need for spatial dependency in the models, we first ran the models without spatial dependency, extracted the Pearson residuals, and plotted these residuals against distance. These plots are called sample variograms[5]. The sample variograms will describe the spatial continuity of the data, informing about spatial patterns (or spatial correlation) present in the model residuals. For all models, the sample variograms indicated spatial autocorrelations, hence the need to include a spatially-structured random effect on grid (as described in Methods). Including spatial dependency in model eq. 1, 3, 4 (change of species richness and CTI) and eq. 5, successfully removed the spatial autocorrelation. However, in model eq. 2 and 4 (change in biotic homogenization) it led to model overfit. Because beta diversity is by definition spatially autocorrelated, it is not surprising that the inclusion of spatial dependency led to overfitting models. Consequently, we did not incorporate spatial dependency in any of the beta diversity models.

Assessing model compatibility

Our analytical approach involved first testing for average change in each community metric (species richness, beta diversity, CTI) over time (Models 1-3, value of each community metric at each grid-cell and time period on a separate row), and then attributing any observed changes to environmental

variables in each grid cell (Model 4, change in community metric in each grid-cell on each row). We believe that this is a sensible and robust approach to both identify the direction and strength of community changes (while also controlling for spatial dependency and sampling effort), and to understand what might be driving these changes. However, this does mean the different models addressing the same community metric contain different error structures. For example, for bird species richness, Model 1 (testing for change) assumed a Poisson distribution in observed species richness, while Model 4 (attributing change) assumed a Gaussian distribution for observed change in species richness. In addition, the different models accounted for observer effort in different ways, with Models 1-2 including observer effort for each grid cell and time period (observer effort not included in Model 3 for CTI, see methods), while Model 4 included change in effort as well as the effort in the earlier time period.

To check for compatibility across these modelling approaches, we used our Models 1-3 to predict community metrics for each grid cell, time period and taxa, and then used a variant of Model 4 in which only observer effort and spatial variables were included to predict change in community metrics for each grid cell and taxa between time periods. We then compared predicted change from Models 1-3 (e.g. predicted value at time 3 minus predicted value at time 1 for long-term change) with predicted change from Model 4. We found that the sign of predicted values (i.e. positive or negative change in species richness, beta diversity or CTI) in each grid cell was broadly consistent across model structures, while the correlation coefficients was also high (Supplementary Table 5). Furthermore, the average difference between model predictions of community change for each metric was low in relation to the range of values of predicted change. This exercise shows that Models 1-3 and Model 4 are compatible in terms of assessing both the direction and magnitude of community change over time using spatial and sampling effort predictors, and therefore the subsequent inclusion of environmental variables for the attribution of change was appropriate. Despite high sign agreement and low relative and absolute differences in predicted values, correlation in CTI across model types was somewhat lower than for species richness and beta diversity. This is understandable: while changes in community composition have commonly been shown to match the general direction of climate warming, rates of community changes are generally only weakly associated with the magnitude of climate warming that has occurred in a specific place[10,11]. CTI models contained only spatial controls (see Methods), and as such we found that predicted change based only on spatial

dependencies were less correlated. Finally, we also note that a main potential issue of the different model structures (Models 1-3 and 4) is related to how the parameter estimates for the predictor variables are interpreted. It was not in our interest to compare the estimates of the spatial and observer-effort variables present across model structures (to each other), while we also do not interpret relative values of parameter estimates of environmental variables from the outputs of Model 4 (e.g. effect of climate vs. effect of land use, or arable vs. urban).

Penalised Complexity priors

Penalised Complexity priors (hereafter, PC priors) are defined as "Probability (*σ > U) = probability α"* i.e., allowing for shrinking the standard deviation (σ) of the random effect towards zero and in this way controlling the smoothness of the spatial structure[12]. The smaller the σ the smoother the trend but if the trend is too smooth it can miss the detail.

For the hyperparameter σ_Y in eq. 3, 4 and 5, we set $\alpha = 0.05$ for all models and tested a range of U values from 0.01 to 1, with the goal to capture as much variance in the response without overfitting the model. As there is no specified methodology to select the best U values for the PC prior, we assessed the choice of the prior calculating the 95% highest posterior density of $\sigma_{Y,}$ (i.e., between -1.96* $\sigma_{Y,}$ and 1.96* σ_Y , where the most of the spatial correlated random effects lies), inspecting residual patterns[5] and calculating the R-square (to guarantee that the inclusion of spatial dependency in the model with the chosen values of U did not overfit). Final values of U are specified in Tables **a** and **b** below (notice U values for plants).

For the Leroux model hyperparameters σ_u and σ_v implemented in model eq. 1, we also used PC priors. For the structured part of the spatial effect $\sigma_{u,}$ we wanted to heavily penalize the spatial structure to ensure that the fixed effects captured as much of the variation as possible. Therefore, we set a very low probability of σ_u being large, i.e., $\alpha = 0.01$, U = 1. For the unstructured part of the spatial effect $\sigma_{v,}$ we wanted a small penalty, and set a high probability of σ_v being large, i.e., $\alpha = 0.2$, U = 1.

Table a – *U* values for PC priors of the σ_Y hyperparameter (random effect with spatial dependent structure) for the long- and short-term change models of SR (species richness) and CTI (community temperature index) of three different taxa in Great Britain – based on model eq. 4 (Methods).

	**SR**		**CTI**	
**Values of U**	*long*	*short*	*long*	*short*
Birds	0.20	0.40	0.10	0.30
Butterflies	0.30	0.45	0.03	0.40
Plants	0.01	0.001	0.10	0.05

Table b – *U* values for PC priors of the σ_Y hyperparameter (random effect with spatial dependent structure) for the local contribution to beta diversity models at three different time periods, and for three different taxa in Great Britain – based on model eq. 5 (Methods).

Local Contribution to Beta Diversity

**Values of U**	*time 1*	*time 2*	*time 3*
Birds	0.10	0.10	0.20
Butterflies	0.20	0.40	0.05
Plants	0.0025	0.002	0.002

References

1. Hill, G. E. & Powers, M. J. Ecomorphs are not species: the case of locally adapted populations of red crossbills. *J. Avian Biol.* **e02896** (2021).

2. Funk, E. R. et al. A supergene underlies linked variation in color and morphology in a Holarctic songbird. *Nat. Comm.* **12**, 6833 (2021).

3. Pescott, O.L., Stroh, P. A., Humphrey, T. A. & Walker, K. J. Simple methods for improving the communication of uncertainty in species' temporal trends. *Ecol. Indic.* 141, 109117 (2022).

4. Blangiardo, M. & Cameletti, M. Spatial and spatio-temporal Bayesian models with R-INLA. (Wiley UK, 2015).

5. Zuur, A. F., Ieno, E. N. & Saveliev, A. A. Beginner's guide to spatial, temporal and spatio-temporal ecological data analysis with R-INLA. Vol. I: Using GLM and GLMM (Highland Statistics Ltd., 2017).

6. Graham, M. H. Factors determining the upper limit of giant kelp, *Macrocystis pyrifera* Agardh, along the Monterey Peninsula, central California, USA. *J. Exp. Mar. Biol.* **218**, 127-149 (1997).

7. Graham, M. H. Confronting multicollinearity in ecological multiple regression. *Ecology* **84**, 2809-2815 (2003).

8. Dormann, C. F., et al. Collinearity: a review of methods to deal with it and a simulation study evaluating their performance. *Ecography* **36**, 27-46 (2013).

9. Auffret, A. G. Historical floras reflect broad shifts in flowering phenology in response to a warming climate. *Ecosphere* **12**(7), e03683 (2021).

10. Zellweger, F. *et al.* Forest microclimate dynamics drive plant responses to warming. *Science* **368**, 772–775 (2020).

11. Auffret, A. G. & Thomas, C. D. Synergistic and antagonistic effects of land use and non-native species on community responses to climate change. *Global Change Biology* **25**, 4303–4314 (2019).

12. Simpson, D., Rue, H., Riebler, A., Martins, T. G. & Sørbye, S. H. Penalising Model Component Complexity: A Principled, Practical Approach to Constructing Priors. *Statistical Science* **32**, 1–28 (2017).
