# Supplementary material for: Anthropogenic climate and land-use change drive short- and long-term biodiversity shifts across taxa
Source: Nat Ecol Evol. 2024 Feb 12;8(4):739–51. doi: 10.1038/s41559-024-02326-7 (PMC11009105; doi:10.1038/s41559-024-02326-7)
Supplement: Supplementary file 2 — Reporting Summary [file 41559_2024_2326_MOESM2_ESM.pdf]

nature portfolio

Corresponding author(s): Alistair Auffret and Teresa Montràs-Janer

Last updated by author(s): Nov 15, 2023

Reporting Summary

Nature Portfolio wishes to improve the reproducibility of the work that we publish. This form provides structure for consistency and transparency in reporting. For further information on Nature Portfolio policies, see our Editorial Policies and the Editorial Policy Checklist.

Statistics

For all statistical analyses, confirm that the following items are present in the figure legend, table legend, main text, or Methods section.

n/a	Confirmed	
☐	☒	The exact sample size (*n*) for each experimental group/condition, given as a discrete number and unit of measurement
☐	☒	A statement on whether measurements were taken from distinct samples or whether the same sample was measured repeatedly
☐	☒	The statistical test(s) used AND whether they are one- or two-sided<br>*Only common tests should be described solely by name; describe more complex techniques in the Methods section.*
☐	☒	A description of all covariates tested
☐	☒	A description of any assumptions or corrections, such as tests of normality and adjustment for multiple comparisons
☐	☒	A full description of the statistical parameters including central tendency (e.g. means) or other basic estimates (e.g. regression coefficient) AND variation (e.g. standard deviation) or associated estimates of uncertainty (e.g. confidence intervals)
☐	☒	For null hypothesis testing, the test statistic (e.g. *F*, *t*, *r*) with confidence intervals, effect sizes, degrees of freedom and *P* value noted<br>*Give P values as exact values whenever suitable.*
☐	☒	For Bayesian analysis, information on the choice of priors and Markov chain Monte Carlo settings
☐	☒	For hierarchical and complex designs, identification of the appropriate level for tests and full reporting of outcomes
☒	☐	Estimates of effect sizes (e.g. Cohen's *d*, Pearson's *r*), indicating how they were calculated

Our web collection on statistics for biologists contains articles on many of the points above.

Software and code

Policy information about availability of computer code

Data collection	No code was used for data collection. All data was downloaded from repositories accessible or upon request.
Data analysis	R version 4.1.1. was used to conduct the analyses, along with the following packages: Sparta (version 0.2.19); betapart (version 1.5.4); adespatial (version 0.3-14); INLA (version 21.02.23)

For manuscripts utilizing custom algorithms or software that are central to the research but not yet described in published literature, software must be made available to editors and reviewers. We strongly encourage code deposition in a community repository (e.g. GitHub). See the Nature Portfolio guidelines for submitting code & software for further information.

Data

Policy information about availability of data

All manuscripts must include a data availability statement. This statement should provide the following information, where applicable:

- Accession codes, unique identifiers, or web links for publicly available datasets
- A description of any restrictions on data availability
- For clinical datasets or third party data, please ensure that the statement adheres to our policy

BTO Bird Atlas data is available on request from (http://www.bto.org/datasets). The data for the Butterflies for the New Millennium recording scheme is available on request from the Butterfly Conservation (https://ukbms.org/request-data). Plant Atlas data is available on request from the Botanical Society of Britain and Ireland (https://bsbi.org/maps-and-data). Historical land-use data are available at http://doi.org/10.5878/9wks-qg91. Modern land cover data was extracted from

March 2021

the 1990 Land Cover Map (https://catalogue.ceh.ac.uk/documents/3d974cbe-743d-41da-a2e1-f28753f13d1e) and 2015 Land Cover Map (https://catalogue.ceh.ac.uk/documents/cb84ee95-01e4-4d55-a33c-380fe01bc58d) at 25-m spatial resolution. Climate data was downloaded from the Met Office, UK website (https://www.metoffice.gov.uk/research/climate/maps-and-data/data/index). The data for the birds' Species Temperature Index were requested from the authors of https://doi.org/10.1038/nclimate1347; for butterflies, Species Temperature Index can be downloaded from GBIF (https://doi.org/10.15468/ug7pft); and for plants, are available from http://doi.org/10.5281/zenodo.1155850

Human research participants

Policy information about studies involving human research participants and Sex and Gender in Research.

Reporting on sex and gender	N/A
Population characteristics	N/A
Recruitment	N/A
Ethics oversight	N/A

Note that full information on the approval of the study protocol must also be provided in the manuscript.

Field-specific reporting

Please select the one below that is the best fit for your research. If you are not sure, read the appropriate sections before making your selection.

☐ Life sciences ☐ Behavioural & social sciences ☒ Ecological, evolutionary & environmental sciences

For a reference copy of the document with all sections, see nature.com/documents/nr-reporting-summary-flat.pdf

Ecological, evolutionary & environmental sciences study design

All studies must disclose on these points even when the disclosure is negative.

Study description

In this study, we use historical and modern datasets of land-use, climate and species observations from national atlas and monitoring schemes, to investigate how baseline and interacting effects of land-use and climate change drive biodiversity changes in British birds, butterflies, and plants over 50+ years (long-term, approx. 1960s-2010s) and 20 years (short-term, 1990-2010s) time-periods. The main dataset consists of 3,715,724 species occurrence records describing the British communities of breeding birds, butterflies, and plants, in the n = 2,670 10-km square grid-cells of the British National Grid. As biodiversity metrics, we use species richness, beta diversity and community temperature index. Our analyses are based on spatially-explicit generalized linear mixed models and spatially-explicit linear mixed models. We use integrated nested Laplace approximation for Bayesian inference. This method approximates Bayesian inference for latent Gaussian models such as the spatial generalized and mixed-effects linear models used in this study, where latent structures in the data need to be captured. Our models include controls for spatial autocorrelation, variation in recorder effort in space and over time, deal with confounding collinearity effects, and control for the effect of microclimatic heterogeneity in moderating climate-driven effects on biodiversity, and for baseline biodiversity conditions.

Research sample

Our study targets the communities of British breeding birds, butterflies and plants at three different time periods (i.e., 1960s, 1990s and 2010s). Specifically, we focus on local-assemblage at the 10-km square grid-cell resolution across Great Britain (i.e., 2,670 grid-cells). We use available datasets. For breeding birds, these data correspond to the atlases from 1968-72; 1988-91 and 2008-11. For plants, to the atlases of 1930-60; 1987-99 and 2000-19 (for which we retrieved the data referring to the period 2010-2019). For butterflies, we use the periods 1970-1974; 1990-94 and 2010-14 from the national recording scheme, Butterflies for the New Millennium, to match the other two taxa. We retrieved a total of 3,715,724 species occurrence records describing the British communities of breeding birds, butterflies, and plants. These available datasets provided us with species-occurrence data for all three taxons, across 2,670 10-km resolution grid-cells over all Great Britain. For each 10-km resolution grid-cell, taxon and time period (1960s, 1990s and 2010s), we calculated three measures of biodiversity: species richness, beta diversity and community temperature index. To calculate the community temperature index metric, we require the species temperature indices. For butterflies, these are available online at "Schweiger, O., Harpke, A., Wiemers, M. & Settele, J. CLIMBER: Climatic niche characteristics of the butterflies in Europe. ZooKeys 367, 65-84 (2014)". For birds and plants, species temperature indices are available upon request at "Devictor V. et al. Differences in the climatic debts of birds and butterflies at a continental scale. Nat. Clim. Change 2: 121-124 (2012)" and "Sparrius, L. B., van den Top, G.G. & van Swaay, C. A. M. An approach to calculate a Species Temperature Index for flora based on open data. Gorteria – Dutch Botanical Archives 40, 073-078 (2018)", respectively. As for environmental data, for each grid-cell, we retrieved 1) land cover data from the available repositories (Land Utilisation Survey of Great Britain, LUSGB, and the 1990 and 2015 1990 Land Cover Maps from the NERC Environmental Information Data Centre); 2) climate data downloadable from the Met Office (UK), available at 5-km spatial resolution and aggregated at 10-km to give the mean values of annual mean temperature and annual total precipitation within years 1965-75, 1985-95 and 2005-15, to match the three time-periods of the biological data; 3) heterogeneity in temperature microclimates available upon request from "Suggitt et al. Extinction risk from climate change is reduced by microclimatic buffering. Nat. Clim. Change 8, 713-717 (2018)."

Sampling strategy

For each taxon (i.e., birds, butterflies, and plants), we included native species (including some taxonomic aggregations and subspecies for plants) that represented stable taxonomic concepts across time from a biological recording perspective (i.e., they have

	been recorded consistently throughout the period of the study). A further description is specified in Methods. In total, 250 species of birds, 55 species of butterflies and 1,587 species of plants were retained for analysis.
Data collection	All data used in this study is freely available from on-line repositories or upon request from authors and organisations, and are described in Methods. Datasets were collated by the authors as specified in Methods.
Timing and spatial scale	This study concerns three time periods, i.e., 1960s; 1990s and 2010s. Observations of breeding birds, butterflies and plants, land-use and climate data are available for each time period and across the 2,670 10-km square grid-cells of the British National Grid included in this study.
Data exclusions	First, we excluded from the analysis those species that did not fulfil the criteria for study inclusion described above (in Sampling strategy) and specified in Methods. Second, because we are using atlas and presence only monitoring data, and we are interested in changes between time periods, for each taxon (birds, butterflies, and plants), we only included grid-cells recorded on both time periods, i.e., 1960s and 1990s for the long-term analysis; 1990s and 2010s for the short-term analysis. This covered all 2,670 grid-cells for the bird dataset; 2,666 grid-cells for the plant data; and 2,013 and 2,022 grid-cells for butterflies at the long- and short-term, respectively.
Reproducibility	All data used in this study is freely available from on-line repositories or upon request from authors and organisations. The results are fully reproducible by following the modelling description in the Methods section.
Randomization	Randomization is not applicable in this study. All data used in this study is already collected and available upon request. However, we had to control for 1) uneven and unknown recorder effort of the monitoring and atlas data collection, to avoid biased results, and 2) spatial dependency, as ignoring it could lead to underestimating the uncertainty of the model predictions. To control for uneven and unknown recorder effort, we estimated for each time-period and taxon, the recorder effort in each focal 10-km grid-cell using the Frescalo approach (further describe in Methods). For the community temperature index analysis, we used a species-threshold cut off similarly to "Macgregor, C.J. et al. Climate-induced phenology shifts linked to range expansions in species with multiple reproductive cycles per year. Nat Commun 10, 4455 (2019)" and "Platts, P.J. et al. Habitat availability explains variation in climate-driven range shifts across multiple taxonomic groups. Sci Rep 9, 15039 (2019)". To account for spatial dependency, we used a Leroux model (which allows the structured part of the spatial residuals to be part of the parameter space, detaching it from the unstructured spatial random effect) and an Intrinsic Conditional Auto-Regressive model (i.e., a random effect with spatial dependent structure - iCAR model) as specified in Methods. We defined the spatial dependency matrix to the eight surrounding grid-cells of each focal grid-cell for both Leroux and iCAR models.
Blinding	Not applicable in the analysis of this study. This study is based on records of birds, butterflies and plants as well as on land-use and climate data that has already been collected and is available on online repositories or upon request.

Did the study involve field work? ☐ Yes ☒ No

Reporting for specific materials, systems and methods

We require information from authors about some types of materials, experimental systems and methods used in many studies. Here, indicate whether each material, system or method listed is relevant to your study. If you are not sure if a list item applies to your research, read the appropriate section before selecting a response.

Materials & experimental systems

n/a	Involved in the study	
☒	☐	Antibodies
☒	☐	Eukaryotic cell lines
☒	☐	Palaeontology and archaeology
☒	☐	Animals and other organisms
☒	☐	Clinical data
☒	☐	Dual use research of concern

Methods

n/a	Involved in the study	
☒	☐	ChIP-seq
☒	☐	Flow cytometry
☒	☐	MRI-based neuroimaging
